# Neuroplastin expression is essential for hearing and hair cell PMCA expression

**DOI:** 10.1007/s00429-021-02269-w

**Published:** 2021-04-12

**Authors:** Xiao Lin, Michael G. K. Brunk, Pingan Yuanxiang, Andrew W. Curran, Enqi Zhang, Franziska Stöber, Jürgen Goldschmidt, Eckart D. Gundelfinger, Maike Vollmer, Max F. K. Happel, Rodrigo Herrera-Molina, Dirk Montag

**Affiliations:** 1 Neurogenetics Laboratory, Leibniz Institute for Neurobiology, Brenneckestr. 6, 39118 Magdeburg, Germany; 2 Department Neurochemistry and Molecular Biology, Leibniz Institute for Neurobiology, Brenneckestr. 6, 39118 Magdeburg, Germany; 3 Department System Physiology and Learning, AG CortXplorer, Leibniz Institute for Neurobiology, Brenneckestr. 6, 39118 Magdeburg, Germany; 4 Research Group Neuroplasticity, Leibniz Institute for Neurobiology, Brenneckestr. 6, 39118 Magdeburg, Germany; 5 Department System Physiology and Learning, Leibniz Institute for Neurobiology, Brenneckestr. 6, 39118 Magdeburg, Germany; 6 Institute of Medical Psychology, Otto-Von-Guericke University Magdeburg, University Hospital, Leipziger Str. 44, 39120 Magdeburg, Germany; 7 Department of Otolaryngology-Head and Neck Surgery, Otto-Von-Guericke University Magdeburg, University Hospital, Leipziger Str. 44, 39120 Magdeburg, Germany; 8 Medical Faculty, Molecular Neuroscience, Otto-Von-Guericke University Magdeburg, University Hospital, Leipziger Str. 44, 39120 Magdeburg, Germany; 9 Centro Integrativo de Biología Y Química Aplicada, Universidad Bernardo O’Higgins, 8307993 Santiago, Chile; 10 Center for Behavioral Brain Sciences (CBBS), 39106 Magdeburg, Germany

**Keywords:** Neuroplastin, Deafness, Hearing, Auditory cortex, Cochlea, Hair cells, Plasma membrane Calcium ATPase, PMCA

## Abstract

Hearing deficits impact on the communication with the external world and severely compromise perception of the surrounding. Deafness can be caused by particular mutations in the neuroplastin (*Nptn*) gene, which encodes a transmembrane recognition molecule of the immunoglobulin (Ig) superfamily and plasma membrane Calcium ATPase (PMCA) accessory subunit. This study investigates whether the complete absence of neuroplastin or the loss of neuroplastin in the adult after normal development lead to hearing impairment in mice analyzed by behavioral, electrophysiological, and in vivo imaging measurements. Auditory brainstem recordings from adult neuroplastin-deficient mice (*Nptn*^*−/−*^) show that these mice are deaf. With age, hair cells and spiral ganglion cells degenerate in *Nptn*^*−/−*^ mice. Adult *Nptn*^*−/−*^ mice fail to behaviorally respond to white noise and show reduced baseline blood flow in the auditory cortex (AC) as revealed by single-photon emission computed tomography (SPECT). In adult *Nptn*^*−/−*^ mice, tone-evoked cortical activity was not detectable within the primary auditory field (A1) of the AC, although we observed non-persistent tone-like evoked activities in electrophysiological recordings of some young *Nptn*^*−/−*^ mice. Conditional ablation of neuroplastin in *Nptn*^*lox/loxEmx1Cre*^ mice reveals that behavioral responses to simple tones or white noise do not require neuroplastin expression by central glutamatergic neurons. Loss of neuroplastin from hair cells in adult *Nptn*^*Δlox/loxPrCreERT*^ mice after normal development is correlated with increased hearing thresholds and only high prepulse intensities result in effective prepulse inhibition (PPI) of the startle response. Furthermore, we show that neuroplastin is required for the expression of PMCA 2 in outer hair cells. This suggests that altered Ca^2+^ homeostasis underlies the observed hearing impairments and leads to hair cell degeneration. Our results underline the importance of neuroplastin for the development and the maintenance of the auditory system.

**Supplementary Information:**

The online version contains supplementary material available at 10.1007/s00429-021-02269-w.

## Introduction

Hearing impairments compromise perception of the surrounding and alter communication with the external world. The recent finding that particular mutations in the neuroplastin gene (*Nptn*) can lead to deafness (Carrott et al. 2016; Zeng et al. 2016) attracted interest in the functions of neuroplastin in the hearing system.

Neuroplastin is a phylogenetically conserved type I glycoprotein belonging to the immunoglobulin superfamily (Langnaese et al. 1997; for review, see Beesley et al. 2014a, b). Polymorphisms in the regulatory region of the human *NPTN* gene correlate with cortical thickness and intellectual abilities in adolescents (Desrivières et al. 2014) and were detected in individuals suffering from schizophrenia (Saito et al. 2007). Recently, the *NPTN* gene has been associated with heart rate (Choy et al. 2018) and lung cancer (Sumardika et al. 2019). Expression of the 65 kD neuroplastin (Np65) isoform derived by alternative splicing from the *Nptn* gene is restricted to brain neurons, whereas the 55 kD isoform (Np55) is present in most cell types but not e.g. in glia (Langnaese et al. 1997). Neuroplastin has been shown to interact as a ligand with the fibroblast growth factor (FGF) receptor (Owczarek et al. 2010), gamma-aminobutyric acid type A (GABAA) receptors (Sarto-Jackson et al. 2012), S100A8/A9, basigin (Sakaguchi et al. 2016; Sumardika et al. 2019), mesencephalic astrocyte-derived neurotrophic factor (MANF) (Yagi et al. 2020), tumor necrosis factor receptor-associated factor 6 (TRAF6) (Vemula et al. 2020), and with itself undergoing homophilic binding (Langnaese et al. 1997; Owczarek et al. 2010). Furthermore, plasma membrane trafficking of monocarboxylic acid transporter 2 (MCT-2, SLC16A7) and XK-related protein 8 (Xkr8) may require neuroplastin as chaperone (Wilson et al. 2013; Suzuki et al. 2016).

Recently, it was shown that the expression of plasma membrane Ca^2+^ ATPases (PMCAs) critically depends on neuroplastin (Bhattacharya et al. 2017; Herrera-Molina et al. 2017) and that neuroplastin engages in tight contact forming functional complexes with PMCAs (Herrera-Molina et al. 2017; Korthals et al. 2017; Schmidt et al. 2017; Gong et al. 2018). In the absence of neuroplastin, PMCA levels are reduced resulting in elevated intracellular Ca^2+^ levels and prolonged decay time to reach resting Ca^2+^ levels after stimulation (Herrera-Molina et al. 2017; Korthals et al. 2017; Schmidt et al. 2017).

The functions of neuroplastin were also addressed by investigation of targeted mouse mutants with specific loss of only the Np65 isoform (Amuti et al. 2016; Li et al. 2019), complete loss of neuroplastin (*Nptn*^*tm1.2Mtg*^, Bhattacharya et al. 2017), conditional loss in glutamatergic neurons (*Nptn*^*lox/loxEmx1Cre*^, Herrera-Molina et al. 2017), or inducible conditional neuron-specific mutants (*Nptn*^*lox/loxPr−CreERT*^, Bhattacharya et al. 2017). These studies proved that neuroplastin is important for multiple pathways and functions. Loss of neuroplastin affects cellular functions as Ca^2+^ homeostasis, long-term potentiation, synapse formation, and more (Bhattacharya et al. 2017; Korthals et al. 2017) resulting ultimately in pronounced deficits including male infertility, depression-like behavior, and learning and memory deficits in *Nptn* mutants (Bhattacharya et al. 2017). Np65 appears to serve particular functions related to cognitive functions and the synaptic balance (Amuti et al. 2016; Li et al. 2019). Strikingly, induced loss of neuroplastin from neurons in adult mice induces retrograde amnesia specifically of associative memories (Bhattacharya et al. 2017).

Recently, the neuroplastin gene was identified as a deafness gene (Carrott et al. 2016). The mutants *audio-1* (I122N), pitch (C315S), and Y219X, generated by *N*-ethyl-*N*-nitrosaurea (ENU) mutagenesis, and two insertional EUCOMM mutants (insertional mutant *Nptn* intron 3 and *Nptn*^*tm1b(EUCOMM)Hmgu*^ with deletion of Ig3 and transmembrane encoding *Nptn* exons 5 and 6) are associated with progressive hearing impairment or deafness (Zeng et al. 2016; Carrott et al. 2016). The common feature of these ENU-generated and EUCOMM mutants is the possibility of expression of truncated and/or instable or altered neuroplastin proteins possibly leading to gain of function and/or negative effects rather than loss of neuroplastin function. Indeed, the extracellular part of neuroplastin or peptides thereof can mimic several functions of neuroplastin (Smalla et al. 2000; Empson et al. 2006; Owczarek et al. 2010, 2011; Herrera-Molina et al. 2014). Furthermore, Zeng et al. (2016) propose the requirement of Np55 expression by outer hair cells needed for cochlear amplification, whereas Carrott et al. (2016) propose Np65 driven synaptogenesis by inner hair cells as necessary for hearing. In addition, these studies disagree on the localization of neuroplastin and the expression of its isoforms in the cochlea.

In this study, we investigate with targeted mutants that cannot express altered or truncated neuroplastin isoforms whether the complete absence of neuroplastin or its loss in adulthood result in deafness and interfere with the central auditory perception and processing. Here, we analyze hearing properties in mice completely lacking neuroplastin, or with conditional loss of neuroplastin in glutamatergic neurons, or with induced neuroplastin loss in adult neurons after normal development. Furthermore, we show that PMCA2 expression in cochlear hair cells depends on neuroplastin, suggesting aberrant Ca^2+^ extrusion in hair cells as the causal mechanism underlying hearing impairments in neuroplastin mutants. Our results show that neuroplastin is required not only during the development of the hearing system but also for the maintenance of hearing capabilities in the adult.

## Materials and methods

### Animals

Neuroplastin-deficient mice *Nptn*^*−*/*−*^ (*Nptn*^*tm1.2Mtg*^) and floxed *Nptn*^*lox/lox*^ mice with neuron-specific inducible PrCreERT (*Nptn*^*lox/loxPrCreERT*^) or with conditional loss in glutamatergic neurons (*Nptn*^*lox/loxEmx1Cre*^) were described (Bhattacharya et al. 2017; Herrera-Molina et al. 2017). A schematic illustration of the mutated neuroplastin alleles and Cre-mediated recombination for these mouse mutants is provided in Suppl. Fig S1A. PrCreERT was activated by daily i.p. injection of 200 µl tamoxifen 10 mg/ml in medical oil for 10 days. All experiments were conducted with adult (> 3 months of age) mice, except when stated otherwise. Mice were kept with a 12-h light/dark cycle and food and water ad libitum. All procedures were in accordance with institutional, state, and government regulations and approved by an ethics committee.

### Behavioral tests

Sex- and age-matched littermate *Nptn*^+*/*+^ mice served as controls for *Nptn*^*−/−*^ mice, and *Nptn*^*lox/lox*^ mice as controls for *Nptn*^*Δlox/loxPrCreERT*^ and *Nptn*^*lox/loxEmx1Cre*^ mice. The experimenter was not aware of the genotype.

Associative learning was assessed by two-way active avoidance in a two-chambered shuttle-box (TSE Systems GmbH) with 10 s of white noise 6 kHz as conditioning (CS) and electrical foot shock as unconditioned stimulus (5 s, 0.3 mA pulsed) delivered after the CS (80 trials/day, 5–15 s of stochastically varied intertrial intervals for 5 consecutive days). Compartment changes during CS were counted as conditioned avoidance reactions. The acoustic startle response to a stimulus (50 ms, 120 dB) and its inhibition by prepulses (PPI) (30 ms; 100 ms before startle stimulus with eight different intensities, 73–94 dB, 3-dB increments, 70 dB white noise background) was analyzed in a startle-box system (TSE Systems GmbH). Habituation (3 min) was followed by two startle trials and in pseudorandom order by 10 startle trials and five trials at each prepulse intensity with stochastically varied intertrial intervals (5–30 s). The maximal startle amplitude was measured by a sensor platform. Fear conditioning was conducted as described (Schilling et al. 2008). Mice were conditioned in an operant chamber (San Diego Instruments) by exploration (2 min) and auditory cue presentation (15 s), followed by a foot shock (2 s, 1.5 mA unpulsed) with one repetition. Twenty-four hours later, mice were placed in the training chamber (context, 5 min) and then returned to their home cage. One hour later, mice were placed in a novel environment (3 min) and then the auditory cue (CS) was presented (3 min). Freezing behavior (immobility) was recorded during all sessions.

### Thalamocortical brain slice recording

The horizontal thalamocortical brain slices (Cruikshank et al. 2002) were prepared from 4–5-month-old animals (*Nptn*^+*/*+^ and *Nptn*^*−/−*^), and then pre-incubated for 1 h in carbogenated (95% O_2_–5% CO_2_) artificial cerebrospinal fluid (ACSF, containing in mM: 110 NaCl, 2.5 KCl, 2.5 CaCl_2_·2H_2_O, 1.5 MgSO_4_·7H_2_O, 1.24 KH_2_PO_4_, 10 glucose, 27.4 NaHCO_3_, pH 7.3) at room temperature. One slice at a time was transferred into a slice recording chamber (Scientific Systems Inc.) and allowed to recover for at least 30 min. The local complex field potentials were obtained from layer 2/3 pyramidal neurons in primary auditory cortex (A1). This intra-cortical pathway was activated with 0.9% NaCl filled glass capillary microelectrodes (3–5 MΩ). The local complex field potentials were recorded and amplified by an extracellular amplifier (EXT-02B, npi, Germany) and digitized at a sample frequency of 20 kHz by AD/DA converter (POWER 1401mkII, CED, UK). The stimulation strength was adjusted to 30–40% of the maximum field potential values.

### In vivo electrophysiology

#### Surgical procedure and recording

Surgery and preparation of mice for the acute experiment (2-month-old *Nptn*^*−/−*^, *n* = 6; 5-month-old *Nptn*^*−/−*^, *n* = 3; 2*-*month-old *Nptn*^+*/*+^, *n* = 6; 5*-*month-old *Nptn*^+*/*+^, *n* = 7) were conducted as described in detail previously (Happel et al. 2010; Deliano et al. 2018; Brunk et al. 2019). Briefly, animals were anesthetized by intraperitoneal injection (0.007 ml/g) of 20% Ketavet (100 mg/ml, Zoetis), 5% xylazine (2% Rompun, BayerVital), and 75% isotonic sodium chloride solution (0.9 g/l, Berlin Chemie).

Based on the vascularization pattern and confirmed by tonotopic mapping, we identified the AC region and implanted a 32-channel single-shank recording electrode (Neuronexus A1x32-50-413; channel impedances between 500 and 800 kΩ) perpendicular to the AC surface.

Local field potentials (LFPs) were recorded in response to pure tones (100 ms, inter-stimulus interval 800 ms, 50 repetitions per tone, 1–32 kHz) at 75 dB SPL. We further presented a pause condition (no sound presentation) and a condensed click condition (Saldeitis et al. 2014) at 75 dB SPL. All stimuli were generated via Matlab (2007), converted into an analog signal by a data acquisition card (NI PCI-BNC2110, National Instruments, Germany), rooted through a controllable attenuator (gPAH, Guger, Technologies, Austria), and amplified by an audio amplifier (Thomas Tech Amp75). Sounds were presented in an acoustic far field environment of 1 m distance between speaker (Tannoy arena KI-8710-32) and the animal.

After implantation, LFPs were recorded for up to 2 h to allow cortical activity to stabilize (Deane et al. 2020). For final data analysis, we chose sets of stimulus repetitions with stabilized responses. Experiments were conducted in an acoustically and electrically shielded recording chamber. Recorded signals were pre-amplified (500×) and bandpass filtered between 3 and 170 Hz by a PBX2 preamplifier, and then digitized at 1 kHz with a multichannel-recording system (Multichannel Acquisition Processor, Plexon Inc.).

### Current source density (CSD) analysis

Based on the tone- and click-evoked local field potentials (LFPs), a one-dimensional current-source density (CSD) profile was derived from the second spatial derivative of the laminar LFP (Mitzdorf 1985):

$$-\mathrm{CSD}\approx \frac{{\delta }^{2}\varnothing \left(z\right)}{{\delta z}^{2}} = \frac{\varnothing \left(z+n\Delta z\right)-2\varnothing \left(z\right)+\varnothing \left(z-n\Delta z\right)}{{\left(n\Delta z\right)}^{2}}.$$

The formula reflects the relation of the field potential (Ø), the spatial coordinate of the cortical laminae (*z*), the inter-channel distance (Δ*z*, 50 μm) and the differentiation grid (*n*). Prior to CSD calculation, LFPs were smoothed using a weighted average of 7 channels (Hamming window, spatial filter kernel size of 300 μm). We extrapolated the edge channels from the recorded LFPs to retain the channels’ information within the corresponding CSDs (Happel et al. 2010).

Based on click-evoked CSD profiles, cortical layers (I/II, III/IV and V/VI) could be identified based on the early granular sink components, that have been shown to reflect direct thalamocortical input into cortical layers III–IV. Cortical layers I/II, and V/VI were assigned accordingly (Happel et al. 2010; Deliano et al. 2018).

By rectifying and averaging the waveforms of the CSDs for each channel (*n*), we can describe the overall evoked cortical activity along the recording electrode without the cancellation effects of sink and source activities. This average rectified CSD (AVREC) provides a temporal waveform of the overall local columnar transmembrane current flow (Givre et al. 1994; Schroeder et al. 1998).

$$\mathrm{AVREC}= \frac{{\sum }_{i=1}^{n}|{\mathrm{CSD}}_{i}|(t)}{n}.$$

Within the resulting AVREC traces, the highest evoked peak amplitude within the first 50 ms after click presentation was determined and compared with the corresponding time window of the pause condition for all animals.

#### Single photon emission computed tomography (SPECT)-imaging

Small animal SPECT-imaging of cerebral blood flow (CBF) was performed in vivo for mapping spatial patterns of neuronal activity as published previously (Bhattacharya et al. 2017; Kolodziej et al. 2014). In short, *Nptn*^*−/−*^ mice (*n* = 7) and control *Nptn*^+*/*+^ mice (*n* = 10) were injected via catheters in the right external jugular vein with 54.47 ± 8.83 MBq of ^99m^Tc hexamethylpropyleneamine oxime (^99m^Tc-HMPAO) with a flow rate of 50 μl/min. After ^99m^Tc-HMPAO-injection animals were anesthetized and scanned using a four head NanoSPECT/CT scanner (Mediso, Hungary). CT and SPECT images were co-registered. CT scans were made at 45 kVp, 177 μA, with 180 projections, 500 ms per projection, and reconstructed with the manufacturer's software (InVivoScope 1.43) at isotropic voxel-sizes of 100 μm. For SPECT-imaging, 24 projections were acquired during a total scan time of 2 h. Axial field of view (FOV) was 20.9 mm. Energy windows were set to the default values of the NanoSPECT/CT (140 keV ± 5%). SPECT images were reconstructed using the iterative algorithm of the manufacturer's software (HiSPECT, SCIVIS, Goettingen, Germany) at isotropic voxel output sizes of 167 μm. SPECT/CT images were manually aligned to a high-resolution MR mouse brain data set (Ma et al. 2005, 2008) based on skull-landmarks of the CTs with the MPI-Tool software (version 6.36, Advanced Tomo Vision, Kerpen, Germany). SPECT brain data were cut out of the SPECT data in Osirix (64-bit, version 5.7.1; Pixmeo, SARL, Bernex, Switzerland) using a whole-brain volume-of-interest (VOI) (Ma et al. 2005, 2008). Brain SPECT data were global mean normalized using the MPI-Tool software. For data analysis, brain ^99m^Tc-distributions in *Nptn*^*−/−*^ mice vs. controls were compared with data sets from a previous study (Herrera-Molina et al. 2017) where *Nptn*^*lox/loxEmx1Cre*^ mice were tested against *Nptn*^*lox/lox*^ controls.

#### Auditory brainstem responses (ABR)

Animals were anesthetized by intraperitoneal injection (0.007 ml/g) of 20% Ketavet (100 mg/ml, Zoetis), 5% xylazine (2% Rompun, BayerVital), and 75% isotonic sodium chloride solution (0.9 g/l, Berlin Chemie) before auditory testings were performed in a single-walled, sound-attenuated, and electrically shielded chamber (Industrial Acoustics, Germany). Stimulus generation and ABR recordings were performed using Tucker-Davis-Technologies (TDT, USA) System 3 hardware. To assess neural sensitivity to acoustic stimulation of the cochleae, ABRs were measured to low rate (21 Hz) acoustic click stimuli (50 µs). Acoustic stimuli were digitally generated by a real-time processor (RX8, 100 k samples/s; Vollmer 2018; Wiegner et al. 2016) and passed through a programmable attenuator (PA5). Stimuli were amplified (AMP84; Thomas Wulf Elektronik) and delivered by a free-field speaker (MF1) located in front of the animal’s head. Prior to recordings, stimulus levels were calibrated to dB pSPL (Burkard 2006) using a probe microphone (46 BE ¼ʺ; G.R.A.S.) and conditioning amplifier (Nexus 2690, B&K). The distance between speaker and microphone was 4 cm, corresponding to the distance between the speaker and the animal’s ear canals. For recordings, subcutaneous electrodes were placed in the posterior midline of the neck (active), the snout (reference), and the back (ground) of the animal. ABRs were recorded using TDT System 3 software (BioSigRP: 21 Hz, 500–1500 repetitions). Recordings were amplified (RA4LI), digitized (RA4PA; 25 k samples/s), and filtered (RX5: 300–3000 Hz, 50 Hz notch filter). The ABR threshold was defined as the lowest stimulation level that evoked a reproducible response according to visual criteria. Thresholds were tested up to a maximum stimulus level of 90 dB.

#### Immunofluorescent staining

For immunohistochemistry, at least three animals for each genotype were analyzed. Adult mice were anesthetized with isoflurane and transcardially perfused with PBS followed by 4% PFA. Cochleae were dissected and post-fixed in 4% PFA overnight. After fixation, PFA was removed and replaced with 0.1 M EDTA for decalcification. Then, the organ of Corti was isolated and processed for immunofluorescence. Cochlear whole mounts were blocked (20% horse serum and 1% Triton X-100 in DPBS, 1 h, room temperature) and incubated with primary antibodies in the same blocking buffer (overnight, 4 °C). Primary antibodies were sheep polyclonal neuroplastin detecting Np65 and Np55 (pan-Np55/65) and goat polyclonal Np65 isoform-specific (1:500, R&D systems), mouse monoclonal pan-PMCA clone 5F10 (1:1000, Abcam), rabbit anti-PMCA1 and rabbit anti-PMCA2 (1:500, Thermo Scientific), rabbit anti-β-III Tubulin (TUJ) (1:1000, Synaptic Systems), rabbit anti-Myosin7a (1:1000, BD Bioscience), mouse anti-Parvalbumin (1:500, Swant). Secondary antibodies were Cy3-conjugated anti-sheep, Cy5-conjugated anti-rabbit or -goat, Alexa Fluor 488 anti-mouse or rabbit (1:1000, Jackson Immunoresearch), phalloidin-iFluor 488 green (1:1000, Abcam), and Sox-2 (E-4) Alexa Fluor 647 (1:300, Santa Cruz). After washing with PBS and briefly with water, the sections were mounted on glass slides with fluoromount g DAPI (Southern Biotech) and were visualized using a Leica SP5 confocal microscope.

#### Quantification of cochlear cells

The quantification of cells was performed as described (Perny et al. 2016). Briefly, for the hair cell analysis, whole mount preparations of cochlea were labeled with antibodies identifying hair cells (Myosin 7a). Only the co-labeled Myosin7a and DAPI-positive hair cells were counted in the preparations. 300–400 HCs were analyzed for each cochlea area (apical, middle, and basal turns). The total number of hair cells was normalized to the length of the basilar membrane and the cell number per 100 µm was calculated. For spiral ganglion neuron (SGN) counting, the density of SGNs was analyzed by counting both TUJ- and DAPI-positive cells in the midmodilar plane (apical, middle, and basal turns). Three nonconsecutive sections were selected from a total of 20 sections for analysis. The number of SGNs from each region was divided by the area of corresponding Rosenthal’s canal.

#### Western blot of brain and cochlear proteins

Whole brain and cochleae were dissected from adult mice and homogenized in 50 mM Tris–HCl buffer (pH 8.1) with protease inhibitor cocktail (Roche). The crude membranes were extracted by adding lysis buffer (50 mM Tris–HCl, 1% Triton X-100 and protease inhibitor cocktail, pH 8.1). Following incubation for 1 h on ice, the supernatant was collected centrifugation at 12,000*g* for 20 min. Samples were denatured with 2× SDS loading buffer for 5 min at 95 °C, analyzed by western blot and probed overnight with anti-Np65 and -Np55 (pan-Np55/65; 1:5000) or goat polyclonal isoform-specific anti-Np65 (1:5000) antibodies (R&D systems). After incubation for 1 h with anti-sheep or -goat IgG secondary antibody (1:8000, Jackson ImmunoResearch) and washing with TBS containing 0.5% Tween 20, the membrane was developed with ECL solution (Intas Chemocan ECL Imaging).

### Statistical analysis

For behavioral experiments and ABR, Statview (SAS Institute, Inc., Cary, NC) was used for analysis of variance, post hoc analysis (Scheffé or Fisher’s protected least significant difference), repeated-measures analysis of variance, and *t* tests. *p* < 0.05 was considered significant. For quantification of cells, analysis of variance with Dunnett’s multiple comparisons test was performed using Prism (version 9, GraphPad Software) In the voxelwise analysis, unpaired *t* tests were calculated to compare brain ^99m^Tc distributions using the MagnAn-software (version 2.4, BioCom, Germany) and uncorrected *p* values as usual in small-animal radionuclide imaging studies (Endepols et al. 2010; Thanos et al. 2013; Wyckhuys et al. 2010). For CSD analysis, a two-sample *t* test between Pause and Click conditions was performed within each group. For local complex field potential analysis, the Mann–Whitney *U* test was used for group comparisons.

## Results

### Hearing deficits and acoustic brainstem responses (ABR) of adult Nptn^***−/−***^ mice

**Fig. 1:**
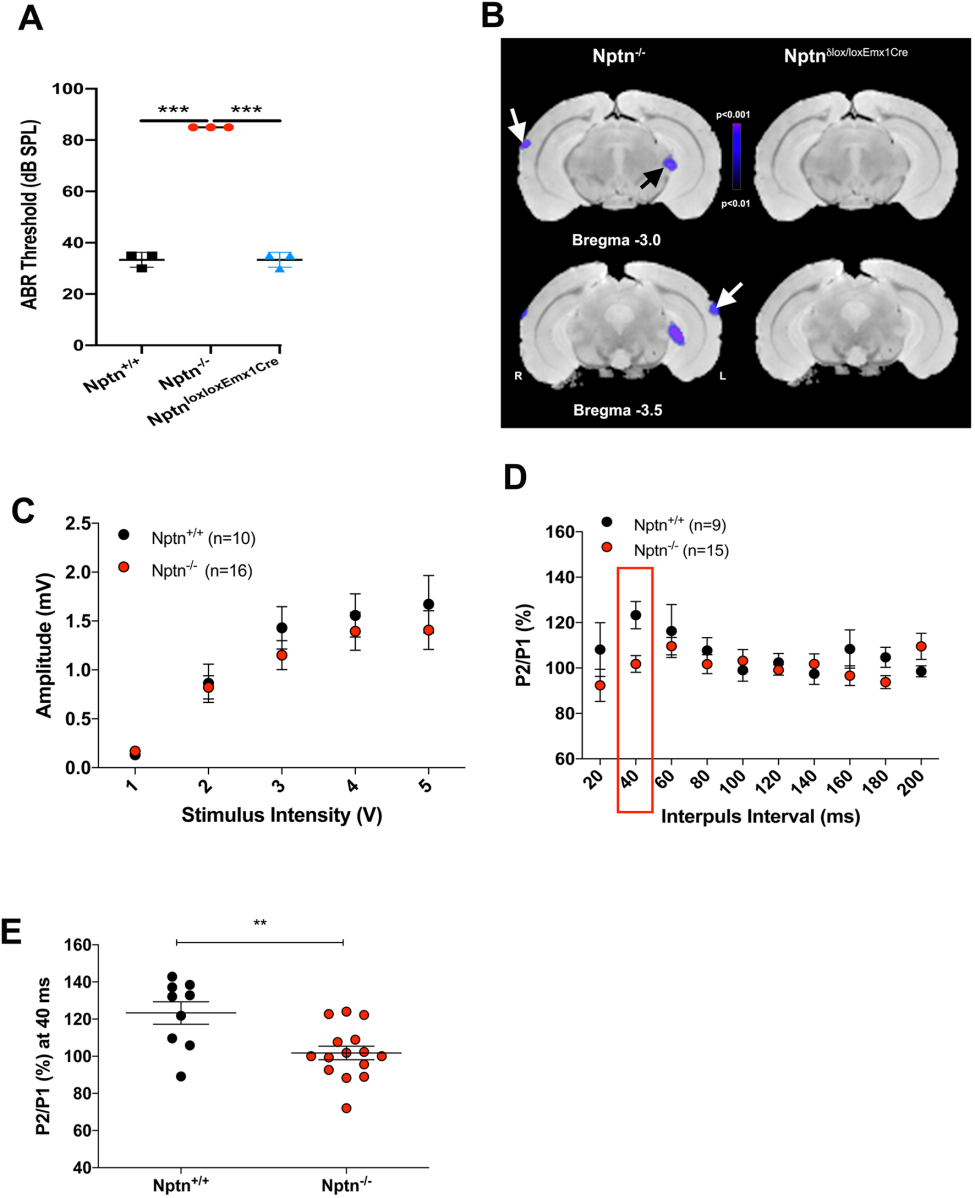
Auditory brainstem responses (ABR) and baseline activity in the auditory cortex of *Nptn*^*−/−*^ and *Nptn*^*loxloxEmx1Cre*^ mice and basal synaptic transmission in *Nptn*^*−/−*^ mice. **A** Comparison of auditory brainstem responses (ABR) to clicks between 4-month-old adult *Nptn*^+*/*+^, *Nptn*^*−/−*^, and 3-month-old *Nptn*^*lox/loxEmx1Cre*^ mice. ABR thresholds in *Nptn*^*−/−*^ (*n* = 3, red circles) were significantly higher in comparison to *Nptn*^+*/*+^ (*n* = 3, black squares) and *Nptn*^*lox/loxEmx1Cre*^ (*n* = 3, blue triangles) mice (one-way ANOVA *F*_(2,6)_ = 480.5; ***p ≤ 0.0001). Thresholds in *Nptn*^*−/−*^ mice reached our criterion for deafness (> 85 dB). **B** Significant changes in cerebral blood flow as determined by SPECT in *Nptn*^*−/−*^ (*n* = 7) and *Nptn*^*loxloxEmx1Cre*^ mice (*n* = 9) as compared to controls (*n* = 10). Shown are maps of *p* values in the range of *p* < 0.01 to *p* < 0.001 overlaid on reference MRIs at two different distances from Bregma. In *Nptn*^*−/−*^ but not in *Nptn*^*lox/loxEmx1Cre*^ mice, blood flow decreases significantly in the auditory system (white arrows point to the AC, black arrow to the medial geniculate body). **C** In brain slices of the AC, input/output (I/O) curves of local complex field potential amplitude revealed no differences in basal synaptic transmission between wild-type *Nptn*^+*/*+^ mice (black, *n* = 10 slices) and *Nptn*^*−/−*^ mice (red, *n* = 16 slices). The data are presented as means ± SEM. **D** Short-term plasticity as evaluated by paired-pulse facilitation was significantly decreased at the 40 ms interval in the AC of *Nptn*^*−/−*^ mice (red, *n* = 15 slices) compared to wild-type *Nptn*^+*/*+^ (black, *n* = 9 slices). The data are presented as means ± SEM. **E** Individual paired-pulse facilitation measurements at the 40 ms interval indicating reduced synaptic release probability characteristics (effect of genotype: Mann–Whitney *U* test: **p < 0.01)

Previously, we observed that adult neuroplastin-deficient mice (*Nptn*^*−/−*^, Suppl. Fig. S1A) hardly reacted with a startle response to a 120 dB stimulus and accordingly did not show PPI of the startle response (Bhattacharya et al. 2017). Here, we confirmed the inability of adult *Nptn*^*−/−*^ mice to process auditory information using a sound associated two-way active avoidance task with white noise as the auditory conditioning stimulus (Suppl. Fig. S1B–D). To further evaluate the possibility of processing deficits in the auditory periphery, we evaluated acoustic brainstem responses (ABR) to transient click stimuli. The hearing threshold of 4-month-old adult *Nptn*^*−/−*^ mice (*n* = 3, 90 dB) was significantly higher than in wild-type littermate controls (*n* = 3, 30–40 dB; *p* < 0.0001) (Fig. 1A) and revealed deafness in *Nptn*^*−/−*^ mice similar as reported for the previously characterized neuroplastin mutants (Carrott et al. 2016; Zeng et al. 2016). In contrast, the ABR in 3-month-old mutants with conditional loss of neuroplastin only in glutamatergic neurons (*Nptn*^*lox/loxEmx1Cre*^ mice, *n* = 3) was similar to control mice (Fig. 1A).

### Auditory cortex activities in Nptn^***−/−***^ mice

The functional behavioral failure to respond to the startle sound and to the white noise-conditioning stimulus and the hearing impairment identified by ABR may also be reflected in AC activities. Therefore, we imaged baseline brain activities by single-photon emission computed tomography (SPECT) of cerebral blood flow. In the resting state with background noise, adult *Nptn*^*−/−*^ mice display substantially and significantly reduced baseline blood flow in the auditory system compared to wild-type littermates. In the AC and in the medial geniculate body, tracer content was more than 15% higher in controls than in *Nptn*^*−/−*^ mice and peak significance values were lower than *p* < 0.01 (Fig. 1B). Significance levels peaked in the upper layers of AC, but blood flow decreased over all layers. Interestingly, *Nptn*^*lox/loxEmx1Cre*^ mice with normal ABR display unchanged resting state activities in the AC (Fig. 1B) and normal startle responses and prepulse inhibition (Suppl. Fig. S1E), indicating that neuroplastin expressed by glutamatergic neurons is neither required for perception nor processing of auditory information.

To investigate whether neuroplastin is involved in signal processing in the auditory cortex, we analyzed synaptic transmission in the primary auditory cortex (A1) by field recording (Fig. 1C) in brain slices from adult mice. The input/output (I/O) curves of local complex field potential amplitude did not differ between wild-type and *Nptn*^*−/−*^ mice indicating monosynaptic transmission. However, with short interpulse intervals of 40 ms, the release probability was significantly (*p* = 0.0087) reduced in *Nptn*^*−/−*^ compared to wild-type mice (Fig. 1D, E), indicating slightly altered release properties. A similar effect was also observed for Schaffer collaterals in the hippocampus of *Nptn*^*−/−*^ mice (Bhattacharya et al. 2017).

To further test the responsiveness of the AC in *Nptn*^*−/−*^ mice to auditory stimuli in vivo, we used multichannel electrophysiological recordings in response to pure tone and click stimulation (75 dB SPL) in anaesthetized animals (Suppl. Fig. S2). Auditory evoked CSD analysis provides a sensitive measure of postsynaptic current flow of synaptic populations and identification of cortical layers (Happel et al. 2010). In young (2-month-old) and adult (5-month-old) *Nptn*^+*/*+^ animals, we found a typical canonical CSD pattern of sensory evoked processing with early synaptic inputs resembling thalamocortical input of layers III/IV and subsequent sink activity across supragranular layers I/II and infragranular V/VI layers (Suppl. Fig. S2A, B). Click evoked AVREC responses were significantly higher than cortical activity during the pause condition in both 2- and 5-month-old mice (Suppl. Fig. S2C, left; two-sample Student’s *t*-test; 2-month-old *Nptn*^+*/*+^, *p* = 0.0075; 5-month-old *Nptn*^+*/*+^, *p* = 0.0323). In comparison, *Nptn*^*−/−*^ mice showed substantially altered cortical response patterns. At the age of 2 months, two of six *Nptn*^*−/−*^ mice demonstrated residual cortical responses to a salient pure tone stimulus (Suppl. Fig. S2A, right). However, both 2- and 5-month-old *Nptn*^*−/−*^ mice showed only weak CSD responses to transient clicks (Suppl. Fig. S2B). Accordingly, the averaged rectified CSD during a 100 ms time window after the click presentation was not significantly different from a pause condition without acoustic stimulation (2-month-old *Nptn*^*−/−*^, *p* = 0.6714; 5-month-old *Nptn*^*−/−*^, *p* = 0.6768; Suppl. Fig. S2C). These findings revealed that *Nptn*^*−/−*^ mice show severely suppressed auditory responses in AC in young animals already at the level of thalamocortical synaptic input. Although this indicates malfunctional auditory processing already at subcortical stages, the effects might still be related to central gain processing deficits (Deane et al. 2020).

### Expression of neuroplastin in the cochlea

**Fig. 2:**
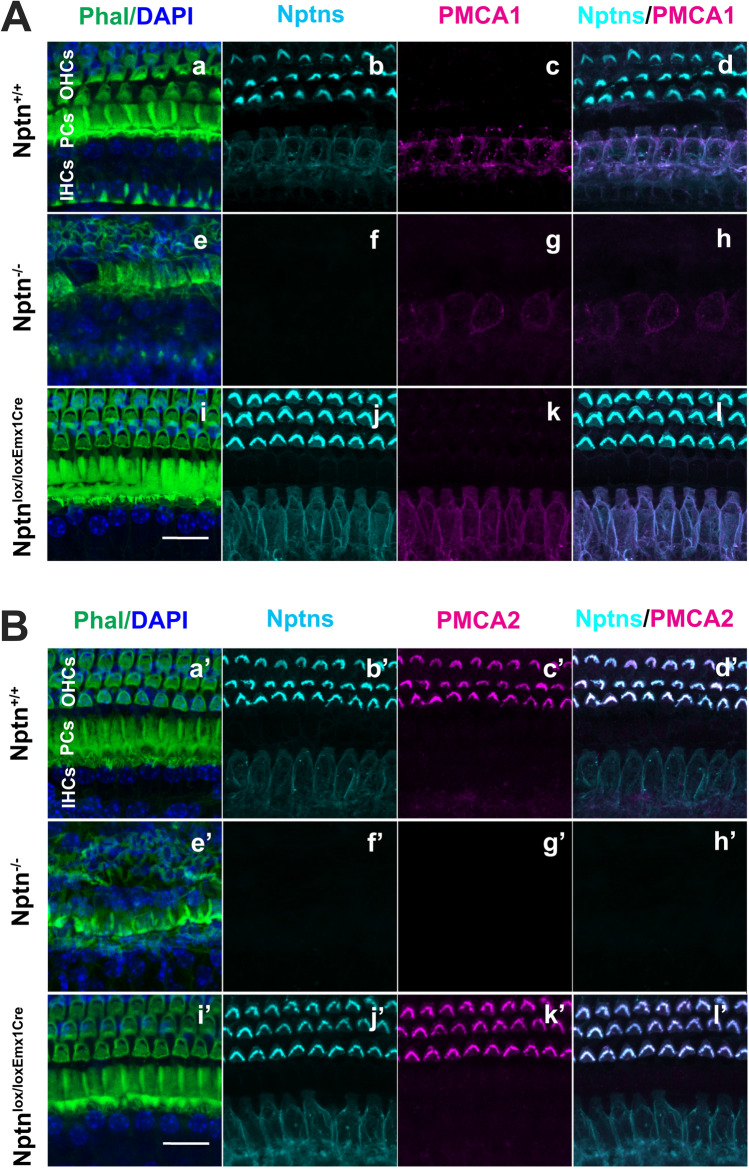
Expression of neuroplastin in inner and outer hair cells of the cochlea. **A**, **B** Cochlear whole mounts of *Nptn*^+*/*+^, *Nptn*^*−/−*^*,* and *Nptn*^*lox/loxEmx1Cre*^ mice were labeled with phalloidin-iFluor 488 green (Phal), DAPI, and antibodies against neuroplastin 55 and 65 (Nptns) and PMCA1 (**A**) or PMCA2 (**B**). Neuroplastin was detected in inner and outer hair cells (b, bʹ, j, jʹ) and colocalized with PMCA1 (c, g, k) in inner (d, l) or with PMCA2 (cʹ, gʹ, kʹ) in outer hair cells (dʹ, lʹ). Scale bar = 15 µm in i and iʹ

**Fig. 3:**
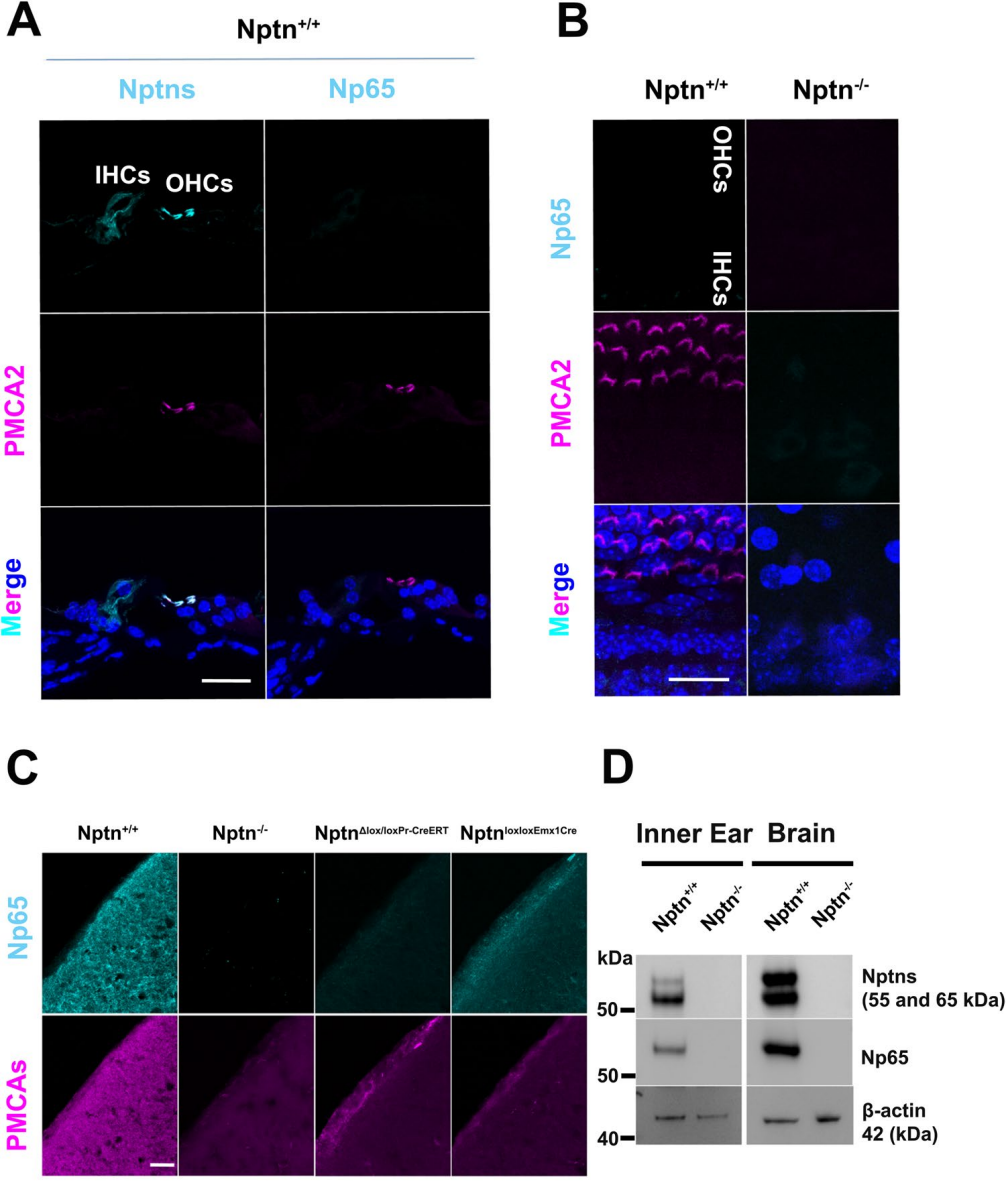
Np65 expressed in the inner ear is not detectable in hair cells. **A** Mid-modiolar cochlear sections from *Nptn*^+*/*+^ mice were labeled with DAPI and antibodies against PMCA2 and against neuroplastin 55 and 65 (Nptns, left) or specific for Np65 (Np65, right). Neuroplastin was detected in inner (IHCs) and outer hair cells (OHCs), but Np65 was not detected. Scale bar = 30 µm. **B** Cochlear whole mounts of *Nptn*^+*/*+^ and *Nptn*^*−/−*^ mice were labeled with DAPI, antibodies against PMCA2, and antibodies specific for Np65. Np65 could not be detected in inner (IHC) or outer hair cells (OHC). Scale bar = 20 µm. **C** Sections from the AC of *Nptn*^+*/*+^, *Nptn*^*−/−*^*,* *Nptn*^*Δlox/loxPrCreERT*^, and *Nptn*^*lox/loxEmx1Cre*^ mice were labeled with antibodies specific for Np65 and antibodies detecting all PMCAs. Np65 was detected in the AC of *Nptn*^+*/*+^ and in reduced quantity of *Nptn*^*lox/loxEmx1Cre*^ mice, but not in *Nptn*^*−/−*^ and *Nptn*^*Δlox/loxPrCreERT*^ mice. Notice that the expression of PMCAs depends on the expression of Np. Scale bar = 30 µm. **D** Western blot analysis of expression of neuroplastin and Np65 in the brain and the inner ear. Antibodies against neuroplastin 55 and 65 (Nptns) and specific for Np65 (Np65) detected the respective isoforms in the brain and the inner ear of *Nptn*^+*/*+^ but not of *Nptn*^*−/−*^ *mice*

Previous work provided limited and conflicting information on the expression of neuroplastin in the cochlea. Carrot et al. (2016) detected Np65 located at the cuticular plate of IHC and OHC and the basolateral region of IHC and could not detect Np55, whereas Zeng et al. (2016) found expression of Np55/65 by spiral ganglia, stereocilia of outer and vestibular hair cells, and the basolateral membrane but were unable to analyze Np65. Therefore, we analyzed expression of neuroplastin in the cochlea of adult wild-type mice in comparison to *Nptn*^*−/−*^ and *Nptn*^*lox/loxEmx1Cre*^ mice (Fig. 2). In wild-type mice, we detected neuroplastin expression by inner and outer hair cells using antibodies against all Np isoforms (Figs. 2A, B, 3A). In contrast, we could not detect any labeling of inner or outer hair cells using antibodies specific for Np65 (Fig. 3A, B), whereas expression of Np65 was easily detected in the AC by immunohistochemistry (Fig. 3C). Western blot analysis of the inner ear of wild-type mice revealed Np65 (Fig. 3D). This indicates, that despite Np65 expression in the inner ear, adult inner and outer hair cells express only Np55. In inner hair cells, neuroplastin was restricted to the cell bodies, whereas it was localized specifically to the stereocilia of the outer hair cells (Fig. 2, Suppl. Fig. S3). Strikingly, this expression pattern matches the known restricted cell body expression of PMCA1 in inner hair cells (Fig. 2A, Suppl. Fig. S3 upper panel) and of PMCA2 in the stereocilia of outer hair cells (Fig. 2B, Suppl. Fig. S3 lower panel) as described by Fettiplace and Nam (2019). The different targeting of neuroplastin and PMCA1/PMCA2 complexes could be caused by differences in the structure of PMCA1 and PMCA2. A Leu-Ile motif in ‘b’-tail splice variants promotes PMCA1b basolateral sorting in inner hair cells, whereas the targeting of PMCA2 depends on the size of the A-site-spliced insert (Grati et al. 2006). Neuroplastin associates with PMCAs to functional complexes permitting Ca^2+^ extrusion (Herrera-Molina et al. 2017; Korthals et al. 2017; Schmidt et al. 2017; Gong et al. 2018), thus, it appears that either the PMCAs direct the differential subcellular localization of neuroplastin in inner and outer hair cells or that neuroplastin determines the localization of the PMCAs, possibly by interaction with further molecules. Indeed, neuroplastin is required for the delivery of PMCA to the cell surface membrane (Schmidt et al. 2017). Furthermore, the absence of neuroplastin resulted in less PMCA1 in inner hair cells and absence of PMCA2 in outer hair cell stereocilia of adult *Nptn*^*−/−*^ mice (Fig. 2). The lack of PMCA2 in outer hair cell stereocilia was already obvious at an age of 18 days (Suppl. Fig. S3 lower panel) before a significant hair cell loss occurs (see below). This suggests that in the absence of neuroplastin, the Ca^2+^ homeostasis in hair cells is compromised leading to the observed hearing deficit. This mechanism is supported by the phenotype of PMCA2-deficient mice, which are completely deaf (Kozel et al. 1998).

**Fig. 4:**
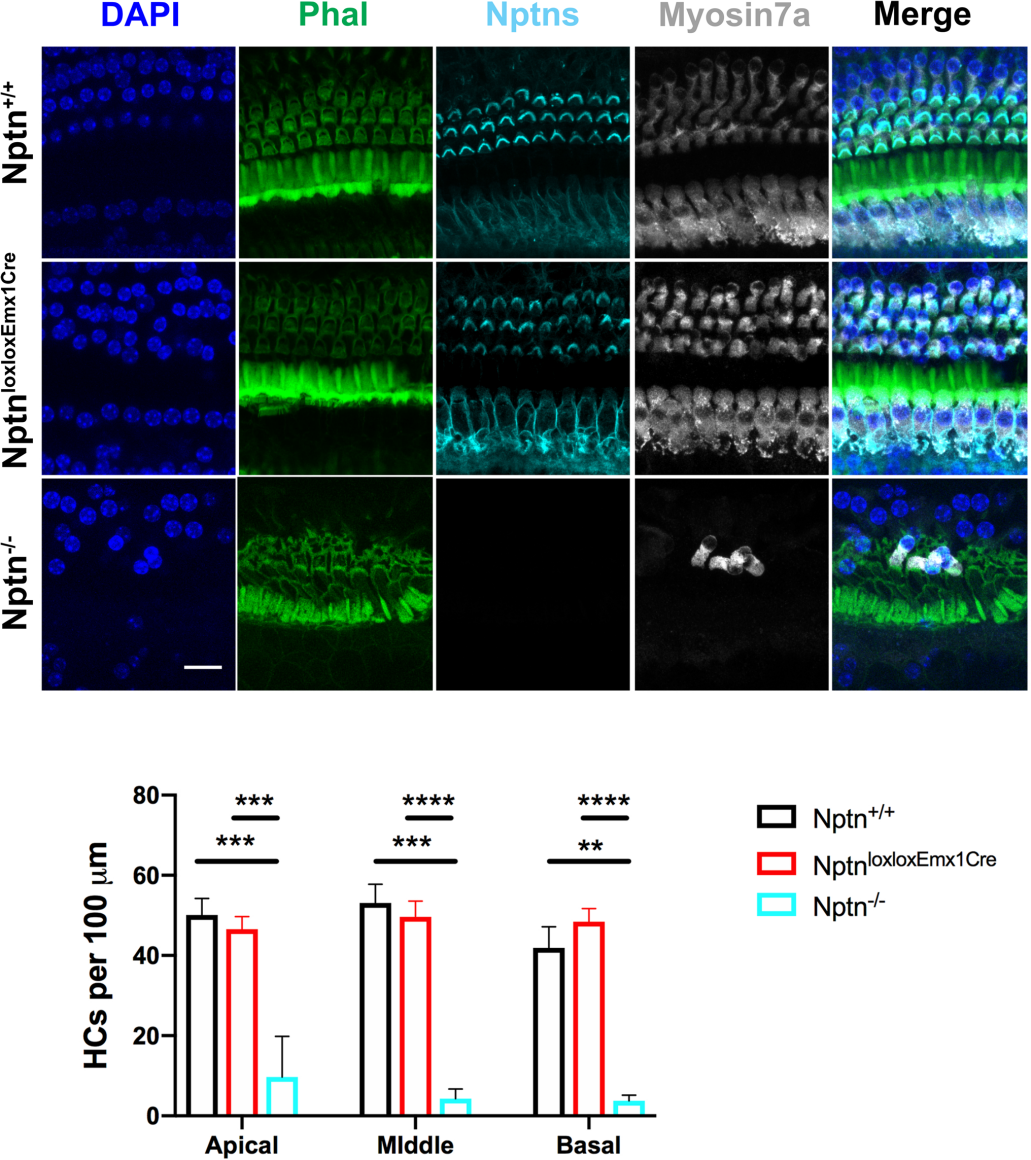
Hair cell degeneration in *Nptn*^*−/−*^ *mice*. Upper part: Representative images of the middle turn of cochlear whole mounts of 4–5-month-old *Nptn*^+*/*+^, *Nptn*^*−/−*^*,* and *Nptn*^*lox/loxEmx1Cre*^ mice labeled with phalloidin-iFluor 488 green (Phal), DAPI, and antibodies against neuroplastin 55 and 65 (Nptns) and Myosin7a. Lower part: Quantification of hair cells identified by Myosin7a in 4–5-month-old *Nptn*^+*/*+^, *Nptn*^*−/−*^*,* and *Nptn*^*lox/loxEmx1Cre*^ mice. The number of hair cells in the apical, middle, and basal areas of the cochlea is significantly reduced in *Nptn*^*−/−*^ (*n* = 5) but not in *Nptn*^*lox/loxEmx1Cre*^ (*n* = 4) in comparison to *Nptn*^+*/*+^ mice (*n* = 3) (1-way ANOVA with Dunnett’s multiple comparisons test, *p ≤ 0.05; **p ≤ 0.01; ***p ≤ 0.001; ****p ≤ 0.0001). Scale bar = 15 µm

**Fig. 5:**
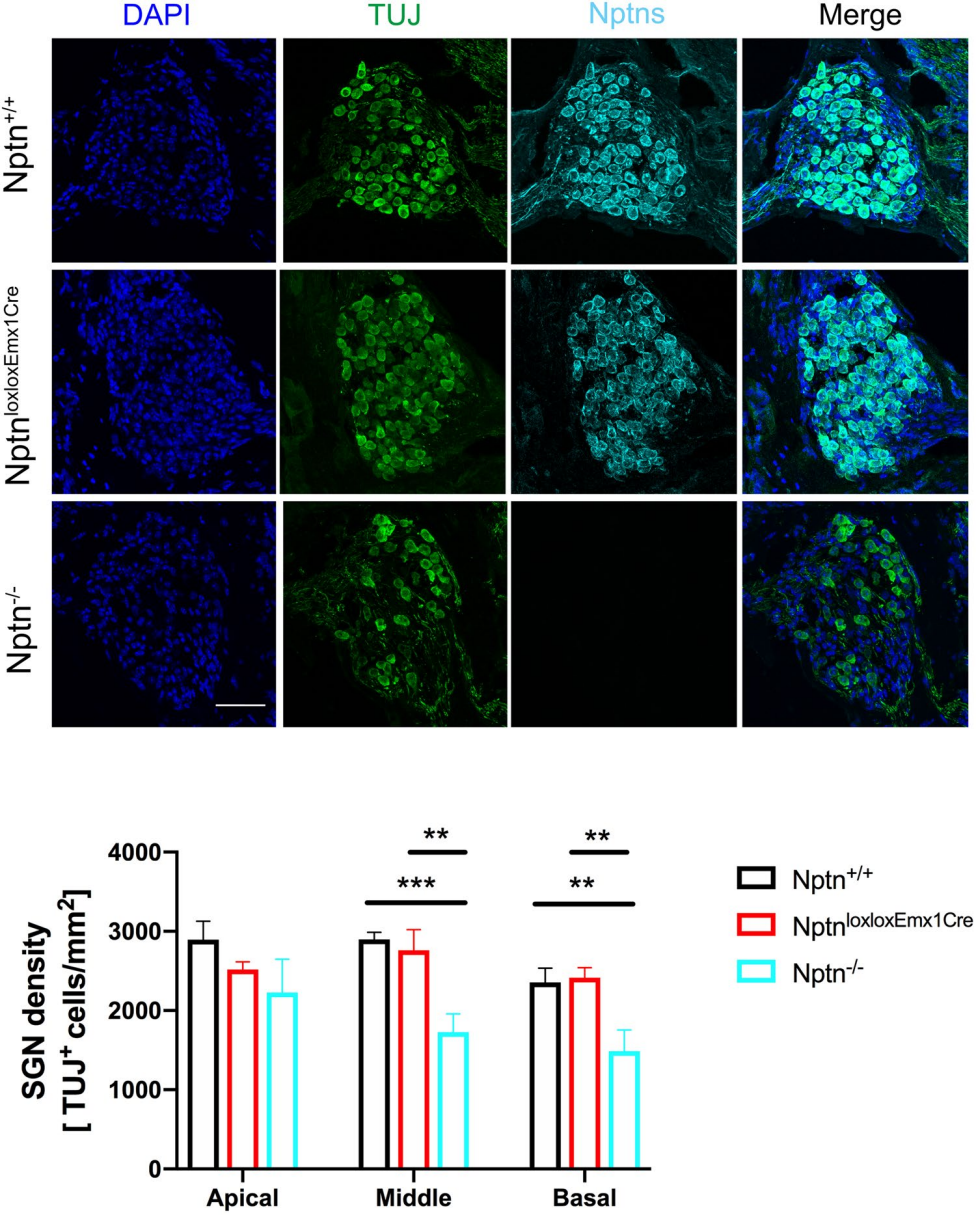
Expression of neuroplastin in spiral ganglion neurons (SGN) of the cochlea. Upper part: Representative immunostaining of the middle part of the Rosenthal’s canal of 4–5-month-old *Nptn*^+*/*+^, *Nptn*^*−/−*^*,* and *Nptn*^*lox/loxEmx1Cre*^ mice labeled with DAPI and antibodies against neuroplastin 55 and 65 (Nptns) and β-III Tubulin (TUJ). Lower part: Quantification of SGN identified by anti-β-III Tubulin staining in 4–5-month-old *Nptn*^+*/*+^, *Nptn*^*−/−*^*,* and *Nptn*^*lox/loxEmx1Cre*^ mice. The number of SGN in the apical area of the cochlea is slightly and in the middle and basal areas significantly reduced in *Nptn*^*−/−*^ (*n* = 4) but not in *Nptn*^*lox/loxEmx1Cre*^ (*n* = 3) in comparison to *Nptn*^+*/*+^ (*n* = 3) mice (1-way ANOVA with Dunnett’s multiple comparisons test, *p ≤ 0.05; **p ≤ 0.01; ***p ≤ 0.001; ****p ≤ 0.0001). Scale bar = 50 µm

At P18 in *Nptn*^*−/−*^, the morphology of the cochlea and in particular the hair cells appeared normal (Suppl. Fig. S3), whereas a considerable loss of hair cells was observed in adult mutants. Therefore, we quantified the hair cell degeneration in the organ of Corti in 4–5-month-old *Nptn*^*−/−*^* mice* using Myosin7a as a hair cell marker. The number of hair cells in the apical, middle, and basal areas of the cochlea was significantly reduced in *Nptn*^*−/−*^ (*n* = 5) but not in *Nptn*^*lox/loxEmx1Cre*^ (*n* = 4) in comparison to *Nptn*^+*/*+^ mice (*n* = 3) (Fig. 4). At this age, the number of spiral ganglion neurons (SGN) in the apical turn of the cochlea was slightly and in the middle and basal turns significantly reduced in *Nptn*^*−/−*^ (*n* = 4) but not in *Nptn*^*lox/loxEmx1Cre*^ (*n* = 3) in comparison to *Nptn*^+*/*+^ (*n* = 3) mice (Fig. 5). Type I SGNs contact the inner hair cells via their peripheral dendrites and relay auditory information to the brainstem via their central axon fibers (for review, Reijntjes and Pyott 2016) whereas type II SGNs contact the outer hair cells (Spoendlin 1969). Both, type 1 and type 2 SGN are detected by the marker beta-III tubulin (TUJ) used by us. However, as type I SGN constitute about 95% and type II SGN constitute about 5% of the total SGN (Ryugo 1992), at least one-third of SGN type I must be lost to account for the observed reduction. Furthermore, the regular layered pattern of supporting cells was disturbed in *Nptn*^*−/−*^mice (Suppl. Fig. S4).

### Neuroplastin expression by glutamatergic neurons is not essential for hearing

In *Nptn*^*lox/loxEmx1Cre*^ mice, Np expression in glutamatergic central neurons is missing (Herrera-Molina et al. 2017), but neuroplastin expression by inner and outer hair cells (Figs. 2, 4) and SGN (Fig. 5) is indistinguishable from wild type. Quantitative analysis of 4–5-month-old *Nptn*^*lox/loxEmx1Cre*^ also did not reveal degeneration of hair cells or SGN (Figs. 4, 5). In agreement, the ABR of *Nptn*^*lox/loxEmx1Cre*^ mice is normal (Fig. 1A). Basal resting brain activities detected by SPECT in *Nptn*^*lox/loxEmx1Cre*^ were reported to differ from control mice in several areas (Herrera-Molina et al. 2017), yet auditory cortical activities were not significantly altered (Fig. 1B). Furthermore, the startle response and its PPI were normal in *Nptn*^*lox/loxEmx1Cre*^ mice (Suppl. Fig. S1E) and *Nptn*^*lox/loxEmx1Cre*^ mice did not show grossly altered cortical response patterns compared to wild-type mice (data not shown). These results show that the absence of neuroplastin from glutamatergic neurons in the brain does not interfere with perception, sensory motor gating, and processing of auditory stimuli. Whether neuroplastin in glutamatergic neurons may be required for more complex functions of the AC remains to be determined.

### Neuronal loss of neuroplastin increases hearing thresholds

**Fig. 6:**
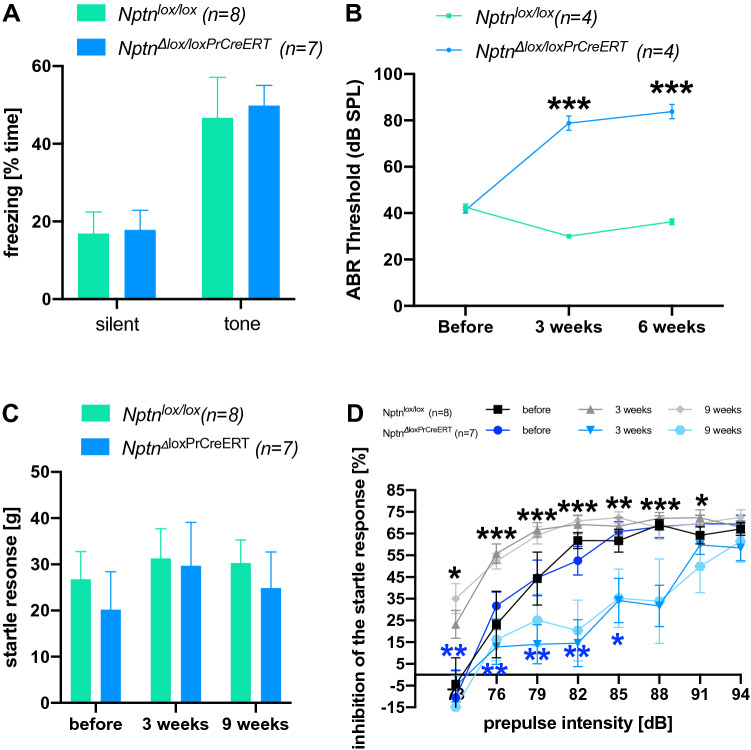
Hearing after ablation of neuroplastin in the adult. **A** Freezing of induced *Nptn*^*Δlox/loxPrCreERT*^ after fear conditioning in a neutral environment. 8 weeks after induction at an age ≥ 3 months, *Nptn*^*lox/lox*^ controls (*n* = 8, green bars) and *Nptn*^*Δlox/loxPrCreERT*^ (*n* = 7, blue bars) mice were subjected to fear conditioning. One day later, the mice were exposed to a neutral environment with different shape, material, and color as the context during fear conditioning. Silent: freezing recorded in the neutral environment without tone; tone: freezing recorded in the neutral environment with the tone paired with foot shock during conditioning. All data are presented as means ± SEM; differences were not significant (1-way ANOVA). **B** ABR of *Nptn*^*Δlox/loxPrCreERT*^ (*n* = 4, blue circles) and *Nptn*^*lox/lox*^ controls (*n* = 4, green squares). Measurements were made at three time points: before induction with tamoxifen (before) at an age ≥ 3 months, 3 weeks post-induction (3 weeks), and 6 weeks post-induction (6 weeks). All data are presented as means ± SEM; differences after induction were significant (1-way ANOVA, ***p ≤ 0.001). **C** Startle response of *Nptn*^*Δlox/loxPrCreERT*^ (*n* = 7, blue bars) and *Nptn*^*lox/lox*^ controls (*n* = 8, green bars). Measurements were made at three time points: before induction with tamoxifen (before) at an age ≥ 3 months, 3 weeks post-induction (3 weeks), and 9 weeks post-induction (9 weeks). All data are presented as means ± SEM; differences were not significant (1-way ANOVA, *p* > 0.05). **D** PPI of the startle response of *Nptn*^*Δlox/loxPrCreERT*^ (*n* = 7, blue colors) and *Nptn*^*lox/lox*^ controls (*n* = 8, grey colors). Inhibition in percent of the startle response without prepulse is presented as means ± SEM. Measurements were made at three time points: before induction with tamoxifen (black squares, dark blue circles), 3 weeks post-induction (triangles, middle grey and middle blue), and 9 weeks post-induction (light grey diamonds, light blue hexagons). Significant differences after induction compared to the respective control are indicated (1-way ANOVA, *p ≤ 0.05; **p ≤ 0.01; ***p ≤ 0.001, black stars: 3 weeks, blue stars: 9 weeks after induction)

**Fig. 7:**
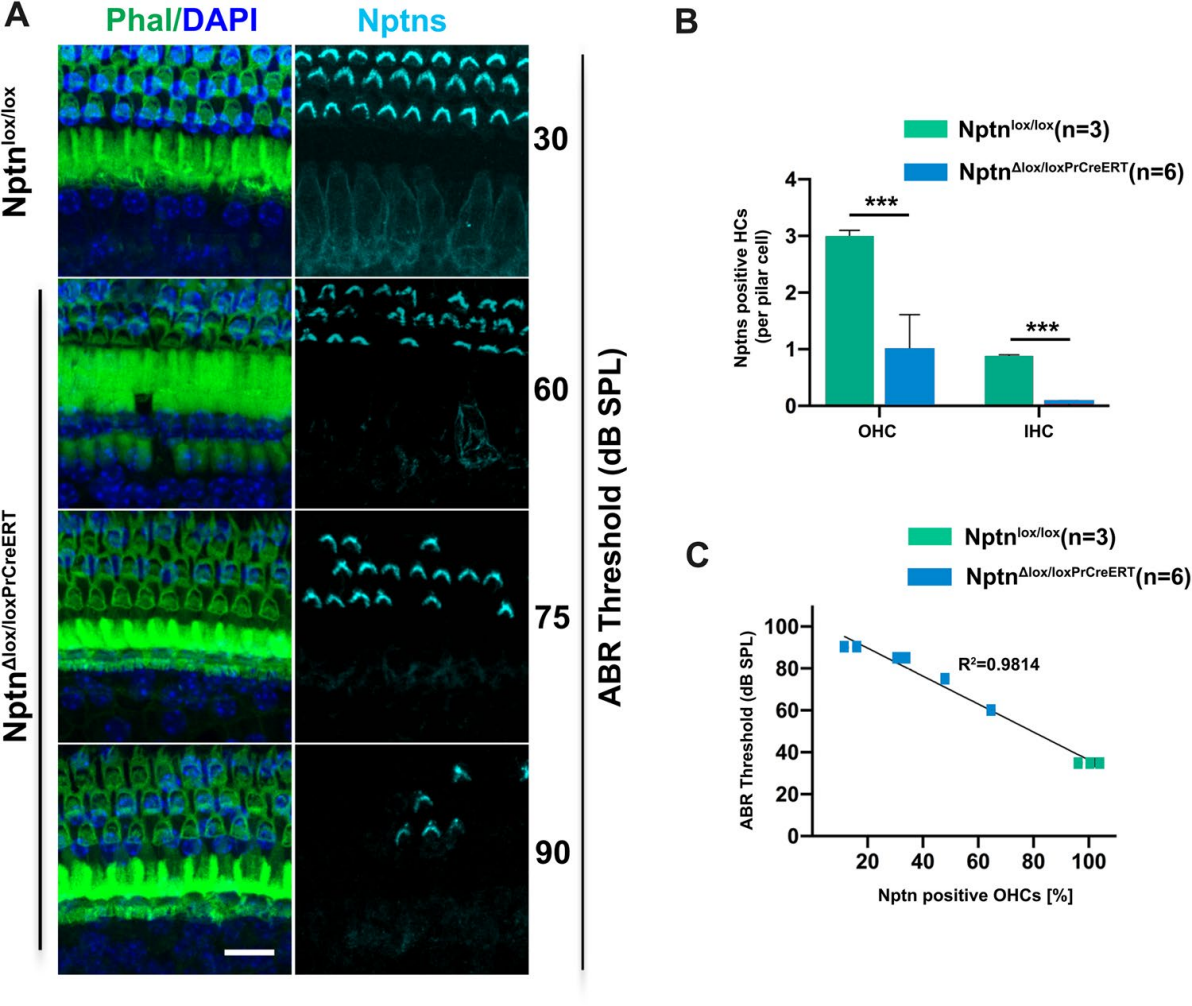
Correlation of ABR thresholds with residual neuroplastin expression in hair cells. **A** Cochlear whole mounts of *Nptn*^*lox/lox*^ mice and *Nptn*^*Δlox/loxPrCreERT*^ mice, that showed different ABR thresholds after induction (indicated on the right), were labeled with phalloidin-iFluor 488 green (Phal), DAPI, and antibodies against neuroplastin 55 and 65 (Nptns). Mice with higher ABR threshold showed less inner and outer hair cells expressing neuroplastin. Scale bar = 15 µm. **B** The number of hair cells (HCs) expressing neuroplastin was significantly reduced after induction in *Nptn*^*Δlox/loxPrCreERT*^ (*n* = 6, blue) compared to *Nptn*^*lox/lox*^ mice (*n* = 3, green). Normalized to pilar cells, the number of outer hair cells (OHC) expressing neuroplastin in stereocilia was strongly reduced (1-way ANOVA; ***p < 0.001) and nearly all inner hair cells (IHC) lost expression of neuroplastin after induction in *Nptn*^*Δlox/loxPrCreERT*^ mice (1-way ANOVA; ***p < 0.001). All data are presented as means ± SEM. **C** ABR thresholds are inversely correlated with the percentage of OHC expressing neuroplastin analyzed in *Nptn*^*Δlox/loxPrCreERT*^ (*n* = 6, blue) and *Nptn*^*lox/lox*^ mice (*n* = 3, green)

To examine, whether neuroplastin is also necessary for maintaining acoustic sensitivity in the auditory system of adult mice, we analyzed *Nptn*^*Δlox/loxPrCreERT*^ mice after inactivating *Nptn* in adult mice (age ≥ 3 months) with the tamoxifen inducible cre-recombinase driven by the prion promoter. Two months after induction, *Nptn*^*Δlox/loxPrCreERT*^ mice respond similar to control mice to the tone presented during fear conditioning with enhanced freezing in a neutral environment (Fig. 6A), which is indicative of sufficient hearing capabilities to respond. Furthermore, when analyzed for memory of fear conditioning trained before induction at an age ≥ 3 months, *Nptn*^*Δlox/loxPrCreERT*^ mice showed 10 weeks after induction *a* reaction similar to control mice to the conditioned tone again indicating at least residual hearing (data not shown). In line with these observations, our previous data showed a normal startle response 6 weeks after induced loss of neuronal neuroplastin in adult mice (*Nptn*^*Δlox/loxPrCreERT*^, Bhattacharya et al. 2017, see ibid Fig. Suppl. 6D + E). However, the PPI of the startle response was reduced at lower prepulse intensities and significantly less at 85 and 88 dB prepulse intensities (Bhattacharya et al. 2017). To investigate, whether *Nptn* ablation in adulthood might lead to progressive hearing deficits we measured ABR and startle response and its PPI over time in *Nptn*^*Δlox/loxPrCreERT*^ and control *Nptn*^*lox/lox*^ mice before and at several time points after induction. In *Nptn*^*Δlox/loxPrCreERT*^ mice that had developed normally and showed normal ABR, startle and PPI before induction at an age ≥ 3 months, the threshold of the ABR increased (Fig. 6B) and the PPI of the startle response with lower prepulse intensities was reduced after neuroplastin ablation (Fig. 6D). However, the startle response itself (Fig. 6C) and PPI with high prepulse intensities remained and did not decline with time (Fig. 6D) in *Nptn*^*Δlox/loxPrCreERT*^ mice. These results suggest a partial hearing loss at lower sound intensities with residual perception of very high stimulus intensities as the consequence of neuroplastin loss in the adult. Indeed, residual neuroplastin expression by inner and outer hair cells after induction in *Nptn*^*Δlox/loxPrCreERT*^ mice correlated with the ABR threshold (Fig. 7). The number of outer and inner hair cells that express neuroplastin was significantly reduced after induction in *Nptn*^*Δlox/loxPrCreERT*^ compared to control *Nptn*^*lox/lox*^ mice (Fig. 7B). Furthermore, the ABR threshold was correlated inversely (*R*^2^ = 0.9814) with the number of neuroplastin expressing outer hair cells (Fig. 7C).

**Fig. 8:**
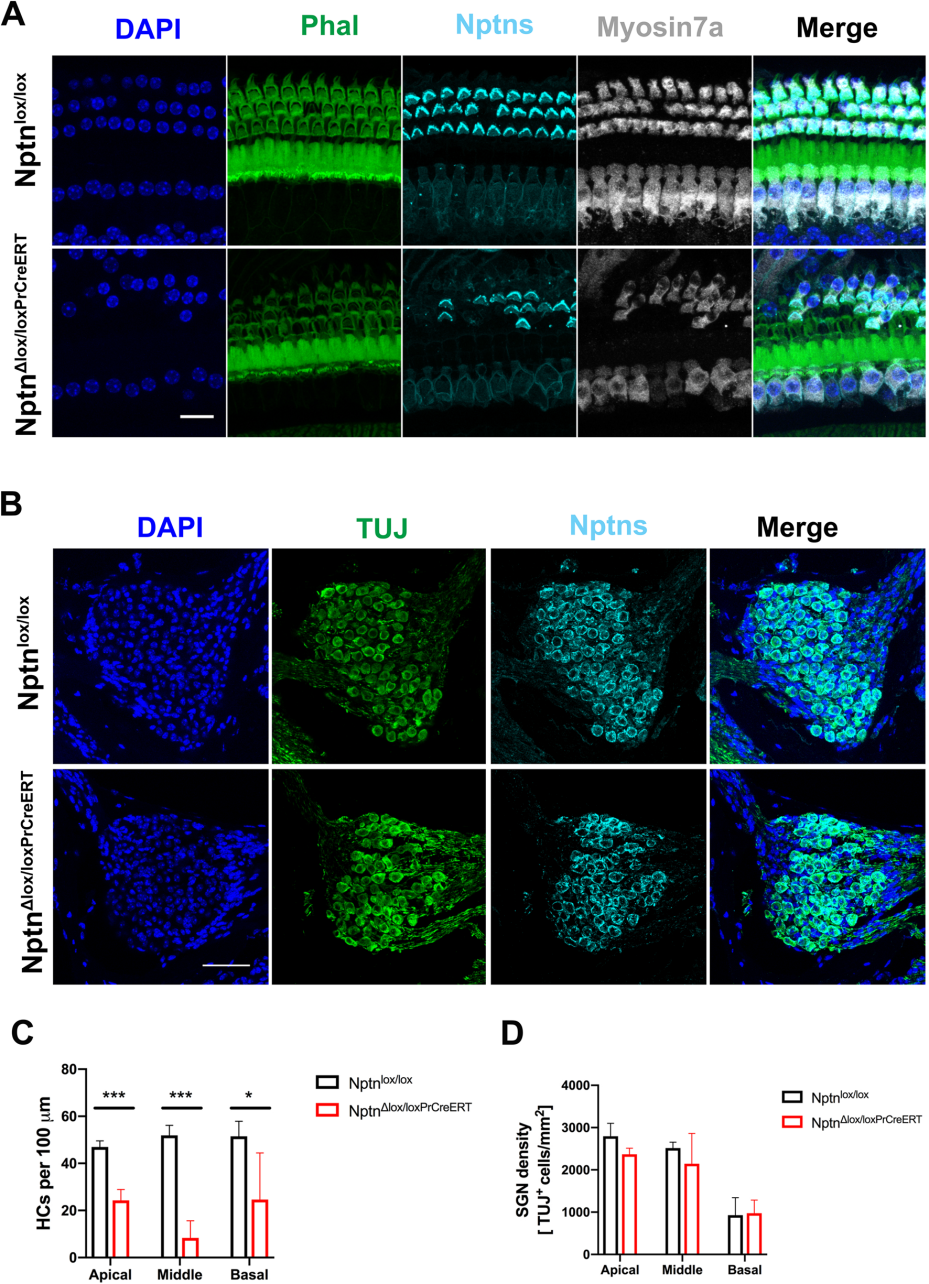
Hair cell degeneration and maintenance of SGN in 9–10-month-old *Nptn*^*Δlox/loxPrCreERT*^ mice 8 weeks after induced loss of neuroplastin. **A** Representative confocal images of the middle turn of the Organ of Corti of *Nptn*^*lox/lox*^ and *Nptn*^*Δlox/loxPrCreERT*^ mice labeled with phalloidin-iFluor 488 green (Phal), DAPI, and antibodies against neuroplastin 55 and 65 (Nptns) and Myosin7a revealing loss of hair cells in *Nptn*^*Δlox/loxPrCreERT*^ mice. Scale bar = 15 µm. **B** Representative immunostainings of the middle area of the Rosenthal’s canal of *Nptn*^*lox/lox*^ and *Nptn*^*Δlox/loxPrCreERT*^ mice labeled with DAPI and antibodies against neuroplastin 55 and 65 (Nptns) and β-III Tubulin (TUJ). SGN still express neuroplastin. Scale bar = 50 µm. **D** Quantification of hair cells identified by Myosin7a in *Nptn*^*lox/lox*^ and *Nptn*^*Δlox/loxPrCreERT*^ mice. The number of hair cells in the apical, middle, and basal areas of the cochlea is significantly reduced in *Nptn*^*Δlox/loxPrCreERT*^ (*n* = 3) in comparison to *Nptn*^*lox/lox*^ (*n* = 4) mice (1-way ANOVA with Dunnett’s multiple comparisons test, *p ≤ 0.05; **p ≤ 0.01; ***p ≤ 0.001). **D** Quantification of SGN identified by β-III Tubulin in *Nptn*^*lox/lox*^ and *Nptn*^*Δlox/loxPrCreERT*^ mice. In *Nptn*^*Δlox/loxPrCreERT*^, SGN still express neuroplastin after induction. The number of SGN in the apical, middle, and basal areas of the cochlea is not affected *Nptn*^*Δlox/loxPrCreERT*^ (*n* = 4) in comparison to *Nptn*^*lox/lox*^ mice (*n* = 3) (1-way ANOVA with Dunnett’s multiple comparisons test)

Quantitative analysis revealed that the number of hair cells in the apical, middle, and basal turns of the cochlea was significantly reduced in *Nptn*^*Δlox/loxPrCreERT*^ (*n* = 3) in comparison to *Nptn*^*lox/lox*^ (*n* = 4) mice 8 weeks after induced loss of neuroplastin in aged mice (Fig. 8A, C). In contrast, the expression of neuroplastin in SGN appeared not to be affected in *Nptn*^*Δlox/loxPrCreERT*^ mice and the number of SGN was similar in *Nptn*^*lox/lox*^ (*n* = 4) and induced in *Nptn*^*Δlox/loxPrCreERT*^ (*n* = 3) (Fig. 8B, D). Expression of neuroplastin in SGN of *Nptn*^*Δlox/loxPrCreERT*^ may be explained by either lack of prion promoter driven Cre expression or eventually by an extremely long half life time of neuroplastin in SGN. In any case, our data indicate that the hearing loss after induction is related to hair cell degeneration but not to SGN reduction.

In conclusion, our data show that in the complete absence of neuroplastin during development the peripheral hair cells fail resulting in deafness and prevention of auditory signal transmission to the AC. Neuroplastin expression by central glutamatergic neurons appears not to be required for auditory perception and processing of simple stimuli (Emx1-promoterCre). When the development of the auditory system initially proceeded normally in the presence of neuroplastin, later loss of neuroplastin in adult hair cells resulted in significant increases in hearing thresholds but not in complete inability to respond to high intensity sounds.

## Discussion

### Auditory deficits in Nptn mutant mice

Using several behavioral paradigms, we have evaluated the capabilities of targeted neuroplastin-deficient mice (*Nptn*^*−/−*^, *Nptn*^*tm1.2Mtg*^) to process auditory information. Despite sufficient pain sensitivity and motoric abilities of *Nptn*^*−/−*^ mice, auditory stimuli did not elicit a startle response or an avoidance reaction towards a tone associated foot shock. Therefore, we suspected an underlying hearing impairment like it was previously reported for other *Nptn* mutants (Zeng et al. 2016; Carrott et al. 2016). Because these previously described ENU-generated and EUCOMM variants may express altered or truncated neuroplastin proteins, the underlying mechanisms could differ from our targeted *Nptn*^*−/−*^ mutant which lacks neuroplastin entirely.

When we determined the auditory thresholds at the auditory periphery/brainstem level, the ABR response in adult *Nptn*^*−/−*^ mice showed very high thresholds above 90 dB revealing that these mice are deaf. In agreement, SPECT imaging of cerebral blood flow as a proxy for neural activity supported functional deficits in the auditory system of *Nptn*^*−/−*^ mice with markedly reduced baseline activities in the AC compared to wild-type littermates. We further investigated whether lack of neuroplastin results in addition to the peripheral hearing deficit in altered signal processing in the auditory cortex. Although, basal synaptic transmission in the primary auditory cortex of *Nptn*^*−/−*^ mice appeared normal with respect to input/output curves determined by field recordings in slices, subtle disturbances of synaptic plasticity in *Nptn*^*−/−*^ mice are suggested by reduced prepulse facilitation at 40 ms intervals. Reduced prepulse facilitation was also described for the hippocampal Schaffer collaterals in *Nptn*^*Δlox/loxPrCreERT*^ at short intervals, whereas *Nptn*^*−/−*^ mice showed enhanced facilitation at larger intervals (Bhattacharya et al. 2017). Based on laminar CSD recordings during presentation of a salient click stimulus, we observed lack of tone-evoked synaptic input in the AC of adult *Nptn*^*−/−*^ mice. Most likely, tone-evoked signals cannot be received and transmitted to the AC for processing. Unlike to muscimol-induced silencing of the AC (Happel et al. 2010; Deane et al. 2020), we did not detect robust and persisting thalamocortical inputs in the granular layers of 2-month-old *Nptn*^*−/−*^ mice in response to pure tone stimulation. Moreover, we did not observe any type of acoustic evoked responses in 5-month-old *Nptn*^*−/−*^ mice. The rudimentary cortical responses in the 2-month-old *Nptn*^*−/−*^ mice (Fig. 3A) might hint at the progression of deafening and an early maladaptive overcompensation of thalamocortical inputs as a result of developmental deafening (Henschke et al. 2018).

We assume that the observed evoked responses in the 2-month-old mice might indicate an initial functional hearing, especially since the rudimentary evoked responses remained nearly consistent during the duration of the experiment. Eventually, the progression of hearing loss leads to the reduction of the thalamocortical inputs in the 5-month-old mice, resulting in a total loss of evoked responses (Chabot et al. 2015).

Our combined data show, that the complete loss of neuroplastin results in deafness already in the auditory periphery that explains the inability of adult *Nptn*^*−/−*^ mice to behaviorally respond to auditory stimuli simply due to peripheral hearing loss. In agreement with the previous studies of other *Nptn* mouse mutants regarding neuroplastin as a deafness gene (Carrott et al. 2016; Zeng et al. 2016), our results underline that deafness can result from the absence of functional neuroplastin during development.

The residual cortical activities detectable in some young *Nptn*^*−/−*^ mice might indicate initial development to a residual activity which is then lost with age leading to deafness in the absence of neuroplastin. This is in line with the suggested progressive hearing deterioration in very young pitch/pitch mutants (Carrott et al. 2016).

The conditional mutant inactivating the neuroplastin gene during development only in Emx1-expressing cells (central glutamatergic neurons) but not in hair cells or SGN of the cochlea shows functional hearing in adulthood. ABR thresholds of *Nptn*^*lox/loxEmx1Cre*^ and wild-type mice do not differ and the behavioral responses to a fear associated tone are similar. Similarly, PPI of the startle response in these conditional mutants is not different from PPI of wild-type mice indicating normal processing and sensorimotor gating. These results indicate that neuroplastin expression by glutamatergic neurons in the brain is not required at any time for a functional auditory system. However, more subtle deficits of hearing or processing of auditory information in these mutants cannot be excluded and might be revealed by more complex hearing tasks. Similarly, neuroplastin expression by glutamatergic neurons is necessary for goal-directed behavior in the water maze (Herrera-Molina et al. 2017). Loss of neuroplastin from adult brain neurons and hair cells of the cochlea in *Nptn*^*Δlox/loxPrCreERT*^ mice led to increased hearing thresholds, but did not completely abolish responses to loud tones. It is well feasible that more complex and demanding auditory tasks may reveal the necessity for neuroplastin expression in the AC.

### Neuroplastin expression in the cochlear hair cells

With our targeted *Nptn*^*−/−*^ mice, that lack both isoforms Np55 and Np65, we cannot resolve, whether both or only one isoform is required for perception and processing of auditory information. Previous studies claimed that Np55 expressed by outer hair cells (Zeng et al. 2016) or Np65 expressed by inner hair cells (Carrott et al. 2016) in the cochlea is necessary for hearing. Our analysis clearly revealed that neuroplastin is expressed by both outer and inner hair cells in the cochlea. Furthermore, the subcellular distribution of Np differs between the hair cells with expression of Np only on the cell bodies of inner hair cells where it is colocalized with PMCA1 and expression of Np only in the stereocilia of the outer hair cells where it is colocalized with PMCA2. Interestingly, loss of Np is always accompanied with loss or reduction of the associated PMCA. By Western blot analysis, we could detect Np65 in the adult inner ear of wild-type mice (Fig. 3D). However, using immunohistochemistry, Np65 was easily detected in the AC but not in inner or outer hair cells (Fig. 3) or in spiral ganglia neurons or in supporting cells (data not shown) confirming that only Np55 is expressed by outer and inner hair cells of the adult cochlea. This is in line with the work of Zeng et al. (2016) but in contrast to the study of Carrott et al. (2016) showing expression of Np65 "localized to the cuticular plate of the IHCs and OHCs and the basolateral region of the IHCs" of the pitch heterozygous mutant. Eventually, the pitch missense mutation may cause this different expression pattern. It would be interesting to investigate the Np65-specific mutants generated by Amuti et al. (2016) for their hearing capacities. Amuti et al. (2016) reported reduced freezing of Np65-deficient mice to a conditioned cue of 87 dB which could indicate reduced hearing capacity and support a role of Np65 for hearing. However, our results indicate that Np65 is not expressed in cochlear hair cells but Np65 may play a role in auditory processing in the auditory pathway.

### Lack of neuroplastin reduces plasma membrane Ca^2+^ ATPases

During development, neuroplastin serves functions as a cell recognition molecule and participates in synapse formation and plasticity (Herrera-Molina et al. 2014; Owczarek et al. 2011). Therefore, structural roles of neuroplastin during development of the ear as proposed by Zeng et al. (2016) and Carrott et al. (2016) are very well feasible. In addition, neuroplastin exerts further critical functions such as the stabilization of Ca^2+^ homeostasis in complexes with PMCA (Herrera-Molina et al. 2017; Korthals et al. 2017; Schmidt et al. 2017; Gong et al. 2018). It has been shown that the hearing system is critically dependent on Ca^2+^ homeostasis and in particular cochlear inner and outer hair cells depend exclusively on PMCA1 and PMCA2 for Ca^2+^ extrusion, respectively (for review, see Fettiplace and Nam 2019). Thus, neuroplastin essentially ensures appropriate levels of PMCAs for optimal Ca^2+^ homeostasis. Our results support a mechanism that requires sufficient Np-dependent expression of PMCA2 in hair cells during development for appropriate Ca^2+^ homeostasis.

When we permitted a normal development and then ablated neuroplastin expression in the neurons of adult animals, hearing thresholds increased with the loss of neuroplastin. Interestingly, we found a linear correlation between the number of outer hair cells expressing neuroplastin and the hearing threshold after ablation of the neuroplastin gene in adult mice. Noteworthy, outer hair cells serve to amplify the auditory signal. These data suggest, that reduced neuroplastin levels lead to a reduction in PMCAs resulting in altered Ca^2+^ homeostasis interfering with auditory reception and signal transduction but residual auditory sensitivity to perceive very loud stimuli remains.

In conclusion, our study shows that functional neuroplastin is essential for the normal development of the auditory system. The complete lack of neuroplastin during development leads to deafness. The processing of auditory click stimuli, pure tones, or white noise does not require expression of neuroplastin by central glutamatergic neurons. Once the auditory system has matured, neuronal neuroplastin is required for normal thresholds in response to acoustic stimulation and only very high-threshold hearing is maintained after the loss of neuroplastin.

## Supplementary Information

Below is the link to the electronic supplementary material.

Supplementary file1 (PDF 26377 KB) **Fig. S1A **Schematic illustration of mutant *Nptn* alleles. By targeted homologous recombination in embryonic stem cells a floxed Nptn allele was generated (Bhattacharya et al. 2017). For *Nptn*^*-/-*^ mice, the floxed neuroplastin gene was inactivated in the germline of mice using CMV-Cre resulting in the *Nptn*<tmΔexon1> (*Nptn*^*-*^) allele. This stable permanent mutant (*Nptn*^*-*^) allele was transmitted through the germline. After backcrossing >10 generations on C57BL/6Crl, *Nptn*^+/-^ mice were intercrossed to obtain *Nptn*^-/-^ mice (Bhattacharya et al. 2017). For *Nptn*^*lox/loxEmx1-Cre*^ mice, mice carrying the homozygous floxed neuroplastin *Nptn*^*lox/lox*^ alleles were crossed with mice carrying the homozygous *Nptn*^*lox/lox*^ alleles plus an Emx1-Cre-transgene (*Nptn*^*lox/loxEmx1-Cre*^) resulting in offspring with complete excision *Nptn*<tmΔexon1> alleles only in cells expressing the Emx1 promoter (Herrera-Molina et al. 2017). In the CNS, neuron specificity is achieved because neuroplastin is expressed only in neurons. For *Nptn*^*lox/loxPrCreERT*^ mice, mice carrying the homozygous floxed neuroplastin *Nptn*^*lox/lox*^ alleles were crossed with mice carrying the homozygous *Nptn*^*lox/lox*^ alleles plus an PrCreERT-transgene (*Nptn*^*lox/loxPrCreERT*^) resulting in offspring in which complete excision to *Nptn*<tmΔexon1> alleles can be induced by tamoxifen only in cells expressing the Prion promoter (Bhattacharya et al. 2017). In the CNS, neuron specificity is achieved because neuroplastin is expressed only in neurons. **Fig.** **S1** **B,** **C,** **D** Two-way active avoidance learning in *Nptn*^*-/-*^ mice. Wild-type control *Nptn*^*+/+*^ mice (n=8, black squares) and *Nptn*^*-/-*^ mice (n=8, red circles) were submitted to a two-way active avoidance learning paradigm (shuttle box) with 80 trials *per* day using white noise as the conditioning stimulus. **B**. number of conditioned runs in percent of total trials. **C**. number of unconditioned runs in percent of total trials. **D**. number of no runs in percent of total trials. All data are presented as means ± SEM; all p values are derived from Scheffé post hoc test after one-way analysis of variance (1-way ANOVA, * p≤0.05; ** p≤0.01; *** p≤0.001). Control mice acquired the task quickly and showed stable performance after 240 trials at day 3 (Suppl. Fig. 1B). In contrast, *Nptn*^*-/-*^ mice did not react to the white noise preceding the foot shock and, therefore, did not learn the sound-associated two-way active avoidance task. As *Nptn*^*-/-*^ mice did react to the foot shock by changing the compartment with unconditioned runs (Suppl. Fig. 1C) and reduced the number of no-runs (Suppl. Fig. 1D), *Nptn*^*-/-*^ mice perceived the foot shock as sufficiently aversive and were motorically able to escape it. Therefore, *Nptn*^*-/-*^ mice either did not perceive the white noise or were unable to associate it with the following foot shock. **Fig.** **S1E** PPI of the startle response of *Nptn*^*loxloxEmx1Cre*^ mice. *Nptn*^*lox/lox*^ controls (n=12, black squares) and *Nptn*^*lox/loxEmx1Cre*^ (n=12, blue circles) were exposed to a startle-eliciting tone (120dB) paired with a prepulse of the indicated intensity. The inhibition of the startle reaction by the prepulse is given in percent of the startle reaction without prepulse. All data are presented as means ± SEM; differences were not significant (1-way ANOVA) indicating normal sensorimotor gating of auditory stimuli. **Fig.** **S2** Sound evoked cortical processing (AVREC and CSD analysis in *Nptn*^*-/-*^ mice. **A**. Left: Example of evoked AVREC traces of a 2-month-old *Nptn*^*+/+*^ mouse for frequencies 1-32 kHz, Pause (P) and Click (C) condition. AVREC trace of corresponding pure tone evoked CSD marked in red. Corresponding CSD pattern of pure tone evoked response is presented next to the AVREC traces of the *Nptn*^*+/+*^ displaying a strong thalamocortical evoked sink activity in layers III/IV after tone onset (dashed line). Right: Evoked AVREC traces of a 2-month-old *Nptn*^*-/-*^ mouse. On the right side, the CSD corresponding to the pure tone evoked AVREC trace labelled in red is shown. A potential tone evoked thalamocortical activity can be found in layers III/IV with a 10-fold lower magnitude compared to the *Nptn*^*+/+*^ animal. **B**. Top row: Click evoked CSD responses of 2- and 5-month-old *Nptn*^*+/+*^ and *Nptn*^*-/-*^ mice. Thalamocortical evoked activity was only found in *Nptn*^*+/+*^ mice whereas *Nptn*^*-/-*^ mice did not show any tone evoked responses. Bottom row: CSD responses of Pause conditions. No tone-evoked responses were found. Displayed are all cortical layers (I/II, III/IV and V/VI) of the AC. **C**. Boxplot group analysis of AVREC peak amplitudes for Pause and Click conditions of all animals. Boxplots are indicating the median, 25% and 75% percentiles and extreme data points (whiskers). An outlier is indicated by a red plus sign. For 2- (n=6) and 5-month-old (n=7) *Nptn*^*+/+*^ animals, differences between pause and click conditions were significant (p=0.0075 and p=0.0323, respectively). In corresponding 2- (n=6) and 5-month-old (n=3) *Nptn*^*-/-*^ animals, no significant differences between pause and click conditions were detected (p=0.6714 and p=0.6768, respectively). P values are derived from two sample t-tests, * p≤0.05. **Fig.** **S3** Lack of PMCA2 expression before hair cell degeneration in *Nptn*^*-/-*^ mice. Representative confocal images of the middle turn of the Organ of Corti of 18 days old *Nptn*^*+/+*^ and *Nptn*^*-/-*^ mice labeled with DAPI, and antibodies against parvalbumin expressed by hair cells, neuroplastin 55 and 65 (Nptns), and PMCA1 (upper panel) or PMCA2 (lower panel). Lack of PMCA2 in outer hair cells is observed in *Nptn*^*-/-*^ mice. Scale bar = 15 µm. **Fig.** **S4** Disorganization of supporting cells in *Nptn*^*-/-*^ and *Nptn*^*Δlox/loxPrCreERT*^mice. Representative confocal images of the middle turn of the Organ of Corti labeled with DAPI, and antibodies against Myosin7a expressed by hair cells, neuroplastin 55 and 65 (Nptns), and SOX2 revealing supporting cells. **Upper** **panel.** 4-5 months old *Nptn*^*+/+*^ and *Nptn*^*-/-*^ mice were investigated. Loss of hair cells and distorted organization of supporting cells in *Nptn*^*-/-*^ mice was observed. **Lower** **panel.** 9-10 months old *Nptn*^*lox/lox*^ and *Nptn*^*Δlox/loxPrCreERT*^ mice eight weeks after induced loss of neuroplastin mice were investigated. Loss of hair cells (most evidently outer hair cells) and slightly distorted organization of supporting cells in *Nptn*^*Δlox/loxPrCreERT*^ mice was observed.

## Data Availability

Available on request with specific Material Transfer Agreements.
