# Supplementary material for: Neuroplastin expression is essential for hearing and hair cell PMCA expression
Source: Brain Struct Funct. 2021 Apr 12;226(5):1533–51. doi: 10.1007/s00429-021-02269-w (PMC8096745; doi:10.1007/s00429-021-02269-w)

SUPPLEMENTARY FIGURE 1

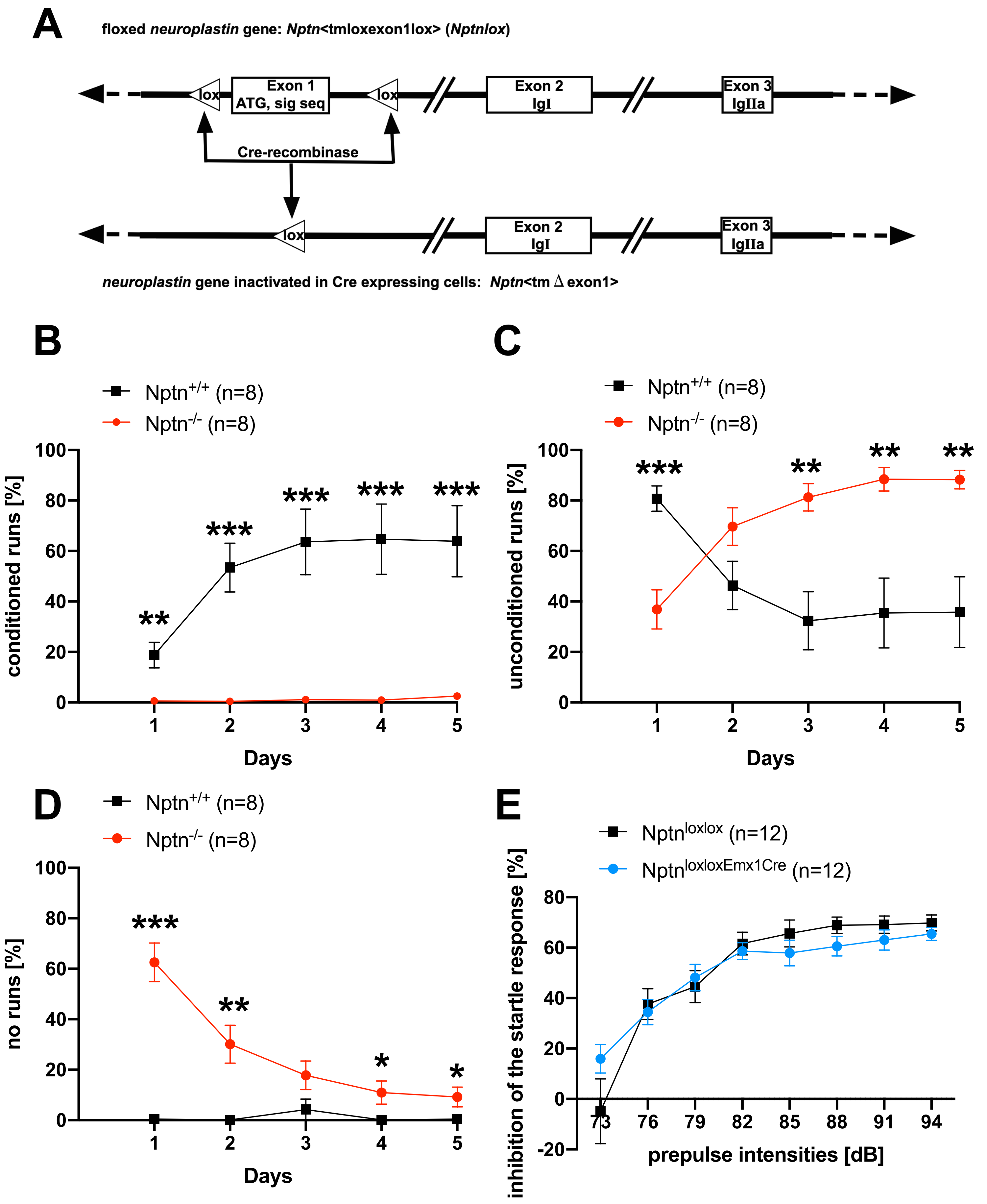

SUPPLEMENTARTY FIGURE 2

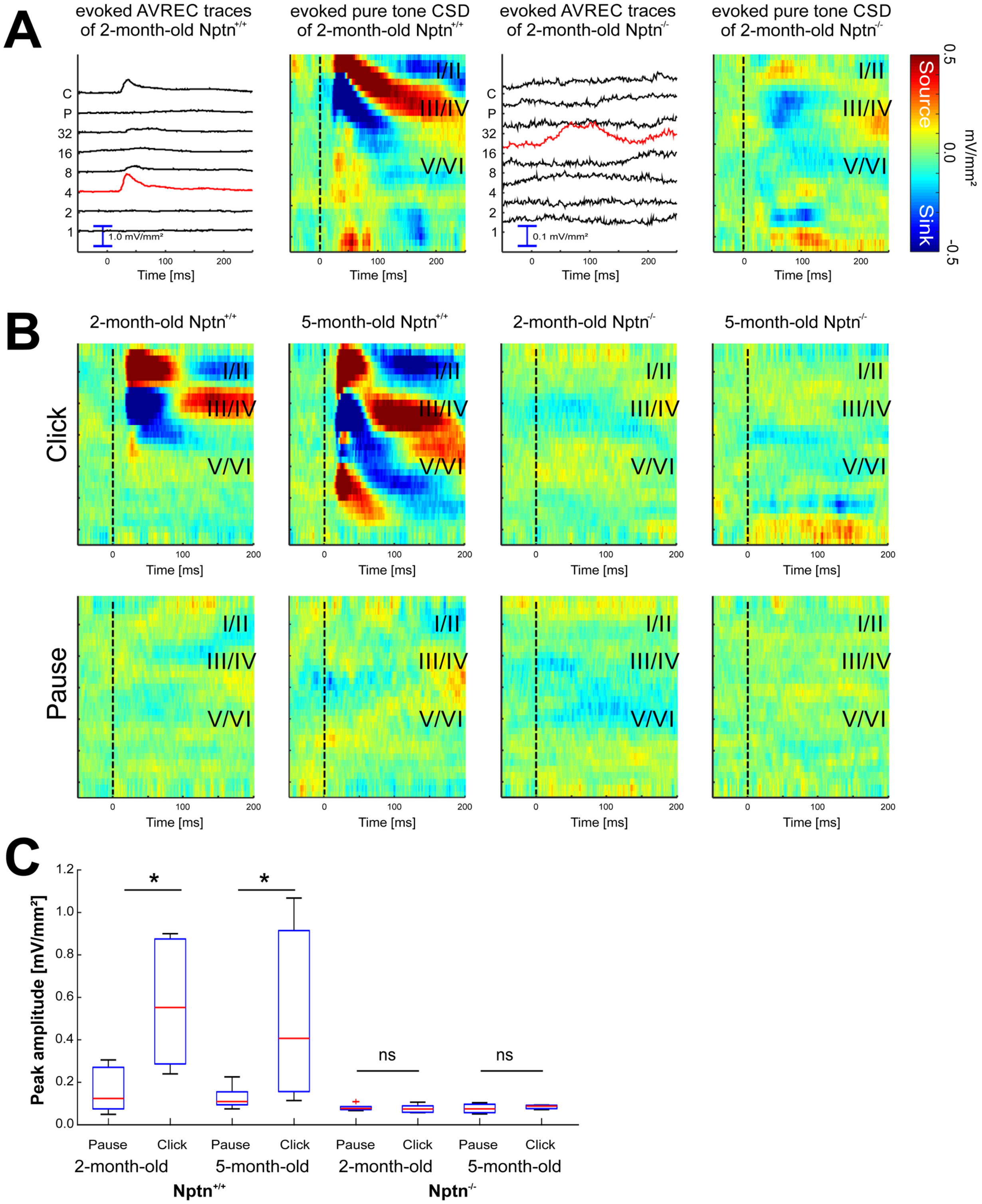

SUPPLEMENTARTY FIGURE 3

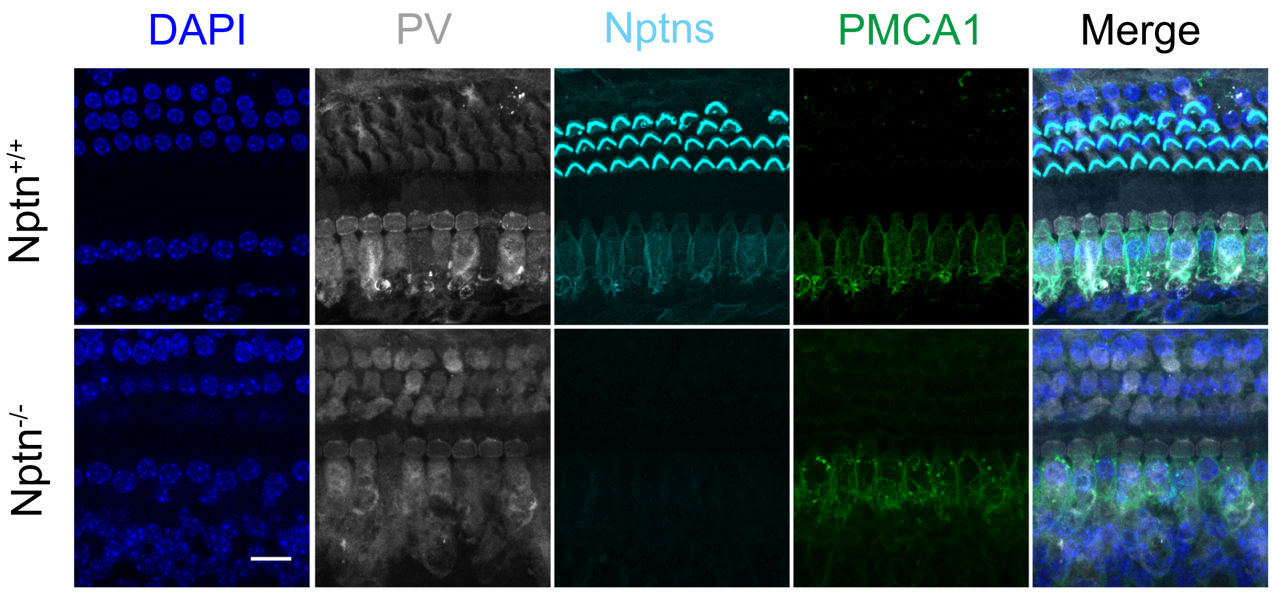

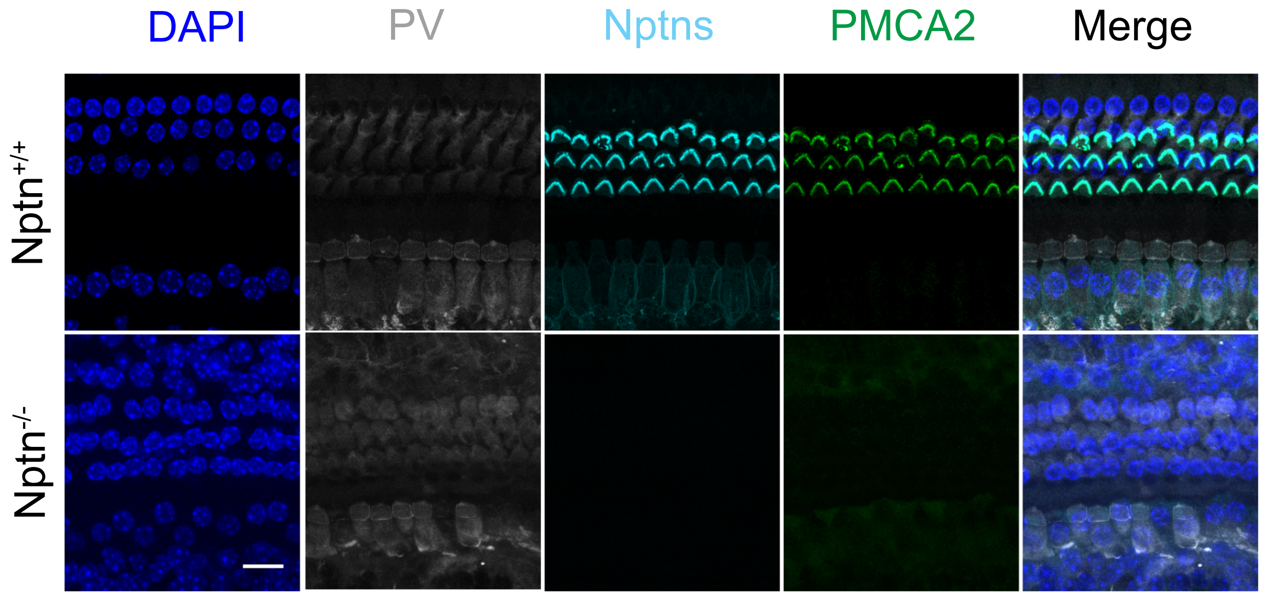

SUPPLEMENTARTY FIGURE 4

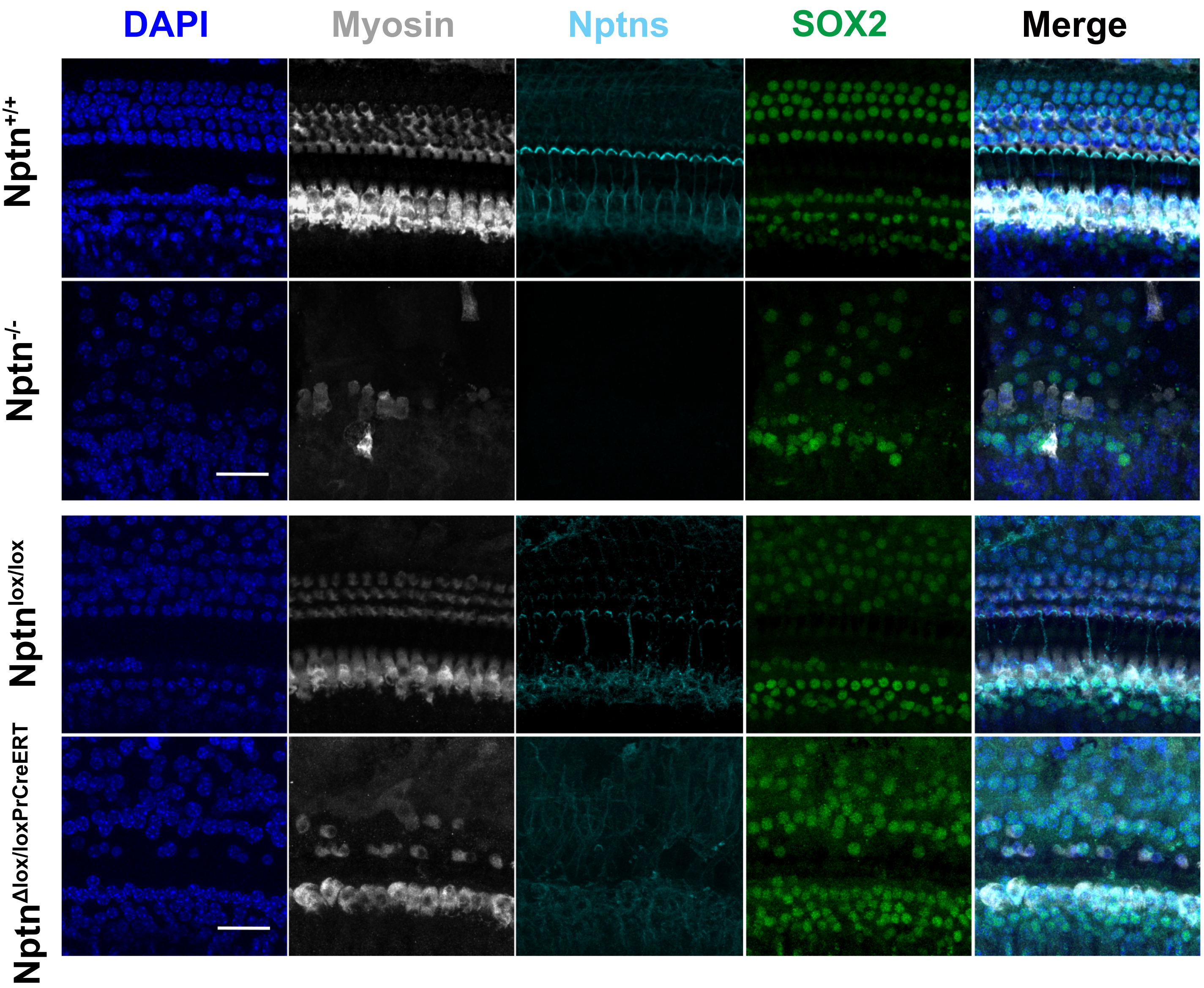

Supplement: Supplementary file 1 — Supplementary file1 (PDF 26377 KB) Fig. S1A Schematic illustration of mutant Nptn alleles. By targeted homologous recombination in embryonic stem cells a floxed Nptn allele was generated (Bhattacharya et al. 2017). For Nptn-/- mice, the floxed neuroplastin gene was inactivated in the germline of mice using CMV-Cre resulting in the Nptn<tmΔexon1> (Nptn-) allele. This stable permanent mutant (Nptn-) allele was transmitted through the germline. After backcrossing >10 generations on C57BL/6Crl, Nptn+/- mice were intercrossed to obtain Nptn-/- mice (Bhattacharya et al. 2017). For Nptnlox/loxEmx1-Cre mice, mice carrying the homozygous floxed neuroplastin Nptnlox/lox alleles were crossed with mice carrying the homozygous Nptnlox/lox alleles plus an Emx1-Cre-transgene (Nptnlox/loxEmx1-Cre) resulting in offspring with complete excision Nptn<tmΔexon1> alleles only in cells expressing the Emx1 promoter (Herrera-Molina et al. 2017). In the CNS, neuron specificity is achieved because neuroplastin is expressed only in neurons. For Nptnlox/loxPrCreERT mice, mice carrying the homozygous floxed neuroplastin Nptnlox/lox alleles were crossed with mice carrying the homozygous Nptnlox/lox alleles plus an PrCreERT-transgene (Nptnlox/loxPrCreERT) resulting in offspring in which complete excision to Nptn<tmΔexon1> alleles can be induced by tamoxifen only in cells expressing the Prion promoter (Bhattacharya et al. 2017). In the CNS, neuron specificity is achieved because neuroplastin is expressed only in neurons. Fig. S1 B, C, D Two-way active avoidance learning in Nptn-/- mice. Wild-type control Nptn+/+ mice (n=8, black squares) and Nptn-/- mice (n=8, red circles) were submitted to a two-way active avoidance learning paradigm (shuttle box) with 80 trials per day using white noise as the conditioning stimulus. B. number of conditioned runs in percent of total trials. C. number of unconditioned runs in percent of total trials. D. number of no runs in percent of total trials. All data are pre [file 429_2021_2269_MOESM1_ESM.pdf]
